# Synthesis and clinical application of new drugs approved by FDA in 2022

**DOI:** 10.1186/s43556-023-00138-y

**Published:** 2023-09-04

**Authors:** Jing-Yi Zhang, Ya-Tao Wang, Lu Sun, Sai-Qi Wang, Zhe-Sheng Chen

**Affiliations:** 1 College of Chemistry and Chemical Engineering, Zhengzhou Normal University, Zhengzhou, 450044 China; 2 First People’s Hospital of Shangqiu, Henan Province, Shangqiu, 476100 China; 3 Henan Engineering Research Center of Precision Therapy of Gastrointestinal Cancer, Zhengzhou Key Laboratory for Precision Therapy of Gastrointestinal Cancer, The Affiliated Cancer Hospital of Zhengzhou University & Henan Cancer Hospital, Zhengzhou, 450008 China; 4 Zhongshan Hospital Affiliated to Dalian University, Dalian, 116001 China; 5 College of Pharmacy and Health Sciences, St. John’s University, Queens, NY 11439 USA

**Keywords:** FDA, NCEs, Synthesis, Clinical applications, Drugs

## Abstract

The pharmaceutical industry had a glorious year in 2022, with a total of 37 new drugs including 20 new chemical entities (NCEs) and 17 new biological entities (NBEs) approved by the Food and Drug Administration (FDA). These drugs are mainly concentrated in oncology, central nervous system, antiinfection, hematology, cardiomyopathy, dermatology, digestive system, ophthalmology, MRI enhancer and other therapeutic fields. Of the 37 drugs, 25 (68%) were approved through an expedited review pathway, and 19 (51%) were approved to treat rare diseases. These newly listed drugs have unique structures and new mechanisms of action, which can serve as lead compounds for designing new drugs with similar biological targets and enhancing therapeutic efficacy. This review aims to outline the clinical applications and synthetic methods of 19 NCEs newly approved by the FDA in 2022, but excludes contrast agent (Xenon Xe-129). We believe that an in-depth understanding of the synthetic methods of drug molecules will provide innovative and practical inspiration for the development of new, more effective, and practical synthetic techniques. According to the therapeutic areas of these 2022 FDA-approved drugs, we have classified these 19 NCEs into seven categories and will introduce them in the order of their approval for marketing.

**Supplementary Information:**

The online version contains supplementary material available at 10.1186/s43556-023-00138-y.

## Introduction

The pharmaceutical industry is constantly evolving, with new drugs being developed and approved by the FDA every year. These new drugs offer hope for patients suffering from various diseases and conditions, and they represent a significant advancement in medical science. In 2022, the FDA approved 37 new drugs that have the potential to revolutionize the treatment of various diseases. Of the 37 new drugs, 20 (54%) new drugs belong to new chemical entity (NCE), and 17 (46%) new drugs belong to new biological entity (NBE) [1]. As shown in Table S1, we summarized the drug names, research & development companies, active ingredients, approval dates, indications, and other information of the 37 new drugs [2]. By analyzing the approved drugs and their indications [3], it can be found that the main therapeutic field is still oncology, and 10 new oncologic drugs were approved (accounting for 27% of all approved drugs). Seven drugs were approved in the field of the central nervous system (accounting for 19% of the total), five drugs were approved in the field of dermatology (14%), four drugs were approved in the field of anti-infective (accounting for 11% of the total), and three drugs were approved in the fields of hematology (accounting for 8% of the total). Two drugs (5%) were approved in ophthalmology and metabolism respectively, and one drug (3% of the total) was approved in the digestive system and cardiomyopathy respectively. Of these 37 new drugs, up to 25 (68%), new drugs were reviewed and approved through the FDA Center for Drug Evaluation and Research (CDER) accelerated approval process. These accelerated approvals are divided into the following categories: fast track, accelerated approval, priority review, and breakthrough therapy. Among them, 18 new drugs received priority review, 9 new drugs obtained fast track, 4 new Drugs received accelerated approval and 13 new drugs obtained breakthrough therapy, including 6 NCEs and 7 NBEs. It is worth noting that 19 (51%) new drugs were approved to treat rare diseases: For example, Enjaymo is used for the treatment of cold agglutinin disease (CAD), a rare, chronic, serious, autoimmune hemolytic anemia disease [4, 5]; Myelofibrosis (MF) is an uncommon condition characterized by abnormalities in the production of blood cells and the presence of fibrosis in the bone marrow [6], and Vonjo received approval to treat primary and secondary MF in adult patients who have experienced a substantial decrease in their platelet levels [7]; Amvuttra was approved by FDA to treat polyneuropathy of hereditary transthyretin-mediated amyloidosis (ATTR), a genetic condition resulting from mutations in the TTR gene [8, 9]. Compared with the 50 drugs approved by the FDA in 2021 (36 NCEs and 14 NBEs), although the number of drugs approved in 2022 is relatively small, there are still some remarkable achievements. For example, Tirzepatide is the first-in-class glucose-lowering drug approved in recent years with a new mechanism of action. In addition to type 2 diabetes, it has shown good potential for treating obesity. The HIV-1 capsid inhibitor lenacapavir can be taken once every six months. Deucravacitinib, a tyrosine kinase 2 inhibitor used to treat psoriasis, the dual-specificity antibody faricimab-svoa for the treatment of macular degeneration, and the antibody–drug conjugate mirvetuximab soravtansine-gynx targeting folate receptor alpha to treat platinum-resistant ovarian cancer have shown good market value.

**Fig. 1:**
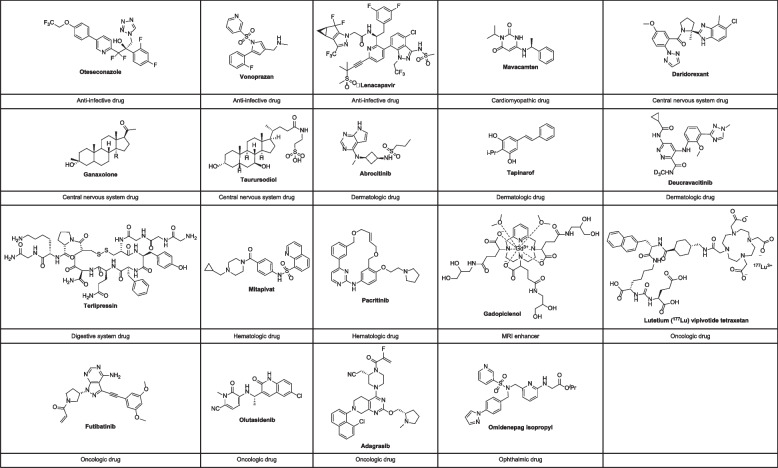
Chemical structures of FDA-approved drugs in 2022

As far as we know, the study of the synthetic methods of new chemical molecules and their mechanisms of action in clinical applications will greatly promote the development of new drugs, and the summary of newly introduced drugs will provide innovative and practical inspiration for new drug discovery [10–20]. Therefore, the purpose of this review is to provide an overview of 19 NCEs approved by the FDA in 2022 (Fig. 1), with a focus on the synthesis of these drugs, their mechanism of action, and their potential benefits and risks. The logical sequence of this review will be to first provide an overview of the new drugs approved by the FDA in 2022 (Table S1), followed by a detailed analysis of the 19 NCEs. Finally, the review will conclude with a summary of the key findings and their implications for healthcare professionals. The review will be of great value to physicians, pharmacists, and other healthcare professionals who are involved in the treatment of patients with the diseases and conditions targeted by these drugs.

## Anti-infective drugs

### Oteseconazole (Vivjoa)

Oteseconazole, developed by Mycovia, was given priority review and approved by the FDA on April 26, 2022, which was sold under the brand name Vivjoa, to prevent recurrent vulvovaginal candidiasis (RVVC) in women with no reproductive potential [21, 22]. As an orally active anti-fungal agent, oteseconazole is a highly potent and selective inhibitor of *Candida albicans* CYP51 with a Ki value of below 39 nM, showing no significant effect on human CYP51 [23–25]. Thanks to the tetrazole moiety, oteseconazole features high selectivity for fungal CYP51 over human P450s, effectively decreasing off-target interactions, which is obviously distinct from previously discovered azole antifungals [26]. Compared with oteseconazole, triazole or imidazole-containing fluconazole or ketoconazole causes significant drug-drug interactions due to their effect on human CYPs [27–29]. By targeting CYP5, oteseconazole blocks the transformation of lanosterol to ergosterol, a sterol necessary for the formation and maintenance of fungal cell membrane integrity, thus exerting antifungal activity toward RVVC-related microorganisms, including Candida dubliniensis, krusei, lusitaniae, albicans, tropicalis, glabrata and parapsilosis [30]. Oteseconazole carries a risk of embryo-fetal toxicity and is therefore not permitted for use in women with reproductive potential [31, 32].

**Fig. 2:**
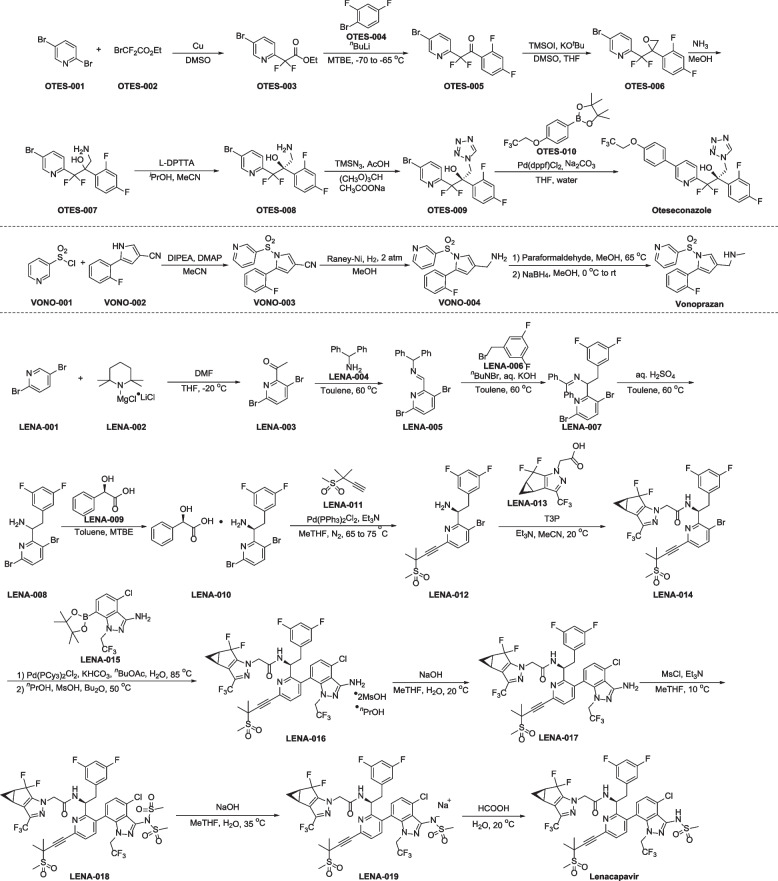
Synthesis of oteseconazole, vonoprazan and lenacapavir

The synthetic method of oteseconazole was reported by Hoekstra, William J. and co-workers (Fig. 2) [33]. The cross-coupling reaction of ethyl bromodifluoroacetate (OTES-002) and 2, 5-dibromopyridine (OTES-001) in the presence of Cu powder in DMSO gives OTES-003. OTES-003 reacts with l-bromo-2,4-difluorobenzene (OTES-004) in methyl *tert*-butyl ether (MTBE), affording OTES-005, which is then subjected to epoxidation reaction in the presence of trimethyl sulfoxonium iodide (TMSOI) and potassium *tert*-butoxide to afford epoxide OTES-006. Subsequent addition reaction affords racemic OTES-007. Compound OTES-007 undergoes chiral resolution with di-*p*-toluoyl-L-tartaric acid (L-DPTTA) in the mix solvents of isopropanol and acetonitrile, followed by the treatment of trimethylsilyl azide, giving tetrazole OTES-009. Finally, OTES-009 undergoes Suzuki–Miyaura reaction with aryl boronic acid OTES-010 to provide coupling compound oteseconazole.

### Vonoprazan, amoxicillin, and clarithromycin (Voquezna)

Vonoprazan was first launched in Japan in February 2015 to treat acid-related diseases and as an adjunct to the eradication of *Helicobacter pylori* [34–36]. As a potassium-competitive acid blocker (PCAB), vonoprazan blocks the secretion of gastric acid mediated by H^+^, K^+^-ATPase, which can be regarded as an alternative to proton-pump inhibitors to treat acid-associated disorders. CYP2C19 genetic polymorphisms show little effect on PCABs, which is obviously different from proton-pump inhibitors [37]. In addition, vonoprazan exhibits 350 times more active than lansoprazole, a proton pump inhibitor, which is attributed to its ability to accumulation in the gastric corpus mucosa, especially in parietal cells [38]. In May 2022, Vonoprazan, in combination with clarithromycin and amoxicillin was approved to treat *Helicobacter pylori* infection [39]. These approvals are based on the safety and effectiveness data of phalcon-hp phase 3 trial, which is the largest registered trial ever conducted in the United States in *Helicobacter pylori*, and 1046 patients were randomly assigned [40]. In the improved intention to treat population, the two vonoprazan treatment regimens showed no worse than lansoprazole triple therapy in patients without clarithromycin or amoxicillin resistant *Helicobacter pylori* strains at baseline [41]. The combination of amoxicillin, vonoprazan and clarithromycin has been reported to result in an eradication rate of *Helicobacter pylori* of about 90% [42, 43].

Among the synthetic methods of vonoprazan [44–46], a relatively simple method starting from pyridine-3-sulfonyl chloride (VONO-001) is exhibited in Fig. 2 [47]. Nucleophilic substitution of VONO-001 with 5-(2-fluorophenyl)-1*H*-pyrrole-3-carbonitrile (VONO-002) gives VONO-003, which then undergoes Raney-Ni promoted reduction reaction to give amine VONO-004. The resulting product VONO-004 is treated with paraformaldehyde and NaBH_4_ generating the desired vonoprazan.

### Lenacapavir (Sunlenca)

Lenacapavir, a first-in-class picomolar inhibitor of HIV-1 capsid protein, is used as a monotherapy, featuring little cross-resistance with clinically used antiretroviral agents and extended pharmacokinetics [48, 49]. The European Commission granted the first worldwide approval of Lenacapavir to treat adults with multidrug-resistant HIV infection on 22 August 2022. On December 22, 2022, it also received FDA approval to treat HIV patients [50, 51]. Lenacapavir exhibits its anti-HIV-1 activity through blocking the viral replication of HIV-1 virus, which is closely related to many processes of viral lifecycle: uptake, assembly, and release [52]. The lenacapavir's difluorobenzyl ring and CPSF6/Nup153 share the same binding pocket, with the benzyl groups of F1417 and F321 overlapping [53]. The crystal structure reveals that six lenacapavir molecules establishes a wide range of interactions with the protein, including cation-π interactions, hydrophobic interactions, and hydrogen bonds, thereby interrupting capsid interactions with CPSF6 and Nup153. In multiple cell lines, in vitro HIV-1 replication inhibition assays show EC_50_ values of ~ 12–314 pM. Lenacapavir exhibits different inhibitory effect at low and high concentrations: it blocks viral nuclear entry at 0.5 nM, while inhibits the reverse transcription and DNA synthesis at 5–50 nM [54].

The process route of lenacapavir is described below in Fig. 2 [55]. The sequence begins with acetylation of commercial 2,5-dibromopyridine (LENA-001) with DMF. This is followed by the condensation with diphenylmethanamine (LENA-004) to access the imine LENA-005. Next, LENA-005 is reacted with 1-(bromomethyl)-3,5-difluorobenzene (LENA-006) affording LENA-007, which is converted to the amine LENA-008 through *N*-deprotection. Racemic LENA-008 undergoes chemical resolution upon treatment with (*R*)-2-hydroxy-2-phenylacetic acid (LENA-009) to obtain single enantiomer salt LENA-010. Subjection of LENA-010 and 3-methyl-3-(methylsulfonyl)but-1-yne (LENA-011) to Sonogashira coupling conditions generates the alkyne LENA-012, and this is followed by condensation with the carboxylic acid LENA-013 in base to provide the amide LENA-014. Suzuki reaction of LENA-014 with the borate ester LENA-015 produces the coupling compound LENA-016. This salt is then removed crystalline alcohol through NaOH followed by nucleophilic substitution with methanesulfonyl chloride yielding LENA-018 with two methanesulfonyl groups. Sequential removal of one of methanesulfonyl group and acidification produce lenacapavir.

## Central nervous system drugs

### Daridorexant (Quviviq)

Daridorexant, the second orexin receptor antagonist after suvorexant, was approved by the FDA on January 10, 2022, for clinical use to treat adult insomnia patients with difficulties of sleep maintenance and/or sleep onset [56, 57]. It was then approved by the European Commission on 3 May 2022, making it the first dual orexin receptor antagonist to be approved for marketing [58]. Daridorexant potently inhibits orexins by working on OX1R and OX2R (Ki = 0.47 and 0.93 nM, respectively), which are wake-promoting endogenous ligands and neuropeptides [59]. Daridorexant is found to decrease overactive wakefulness. Daridorexant has been reported to improve daytime functioning and sleep in insomnia patients [60]. Before the approval of daridorexant, two orexin receptor antagonists have been marketed, including Mercer's suvorexant (approved in 2014) and Eisai's lemborexant (approved in 2019) [61]. But neither has fared well in the market so far.

**Fig. 3:**
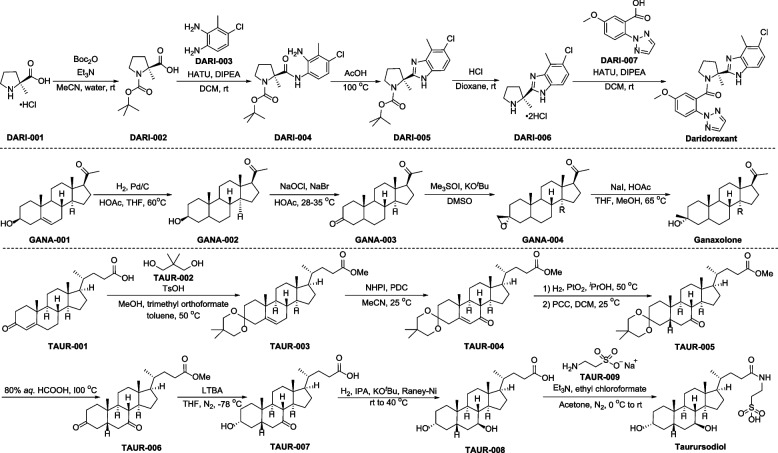
Synthesis of daridorexant, ganaxolone, and taurursodiol

The synthesis of daridorexant is outlined in Fig. 3 [62]. 2-Methyl-L-proline hydrochloride (DARI-001) is treated with di-*tert*-butyl pyrocarbonate (Boc_2_O) in 1/1 mixture solvents of MeCN and water, giving *N*-Boc protection product DARI-002. Treatment of DARI-002 with 6-chloro-2,3-diaminotoluene (DARI-003) under condensation reaction conditions furnishes amide DARI-004. Next, intramolecular condensation of DARI-004 under 100 °C provides DARI-005, which then undergoes *N*-Boc deprotection and condensation with 5-methoxy-2-(2*H*-1,2,3-triazol-2-yl)benzoic acid (DARI-007) to provide daridorexant.

### Ganaxolone (Ztalmy)

Developed by Marinus, Ganaxolone was granted FDA approval as the inaugural treatment specifically indicated for seizures in cyclin-dependent kinase-like 5 deficiency disorder (CDD) [63, 64]. Ganaxolone is one of the metabolites of progesterone from 3β-methylation of allopregnanolone [63]. Ganaxolone, a new class of neuroactive steroids, is effective positive allosteric modulators of γ-aminobutyric acid type A (GABAA) receptors [65], which has specific and potent efficacy, like its endogenous counterparts [66]. Ganaxolone exerts its effect by binding to one specific allosteric binding site of GABAA, which is different from that of benzodiazepine [67]. It is characterized by analgesic, sedative, anxiolytic, hypnotic, anticonvulsant, and anesthetic properties [68]. It is important to note that antiepileptic medications may increase the risk of suicidal ideation and suicidal behavior, and caution should be taken when considering treatment with ganaxolone [69–71].

To this day, several synthetic methods of ganaxolone have been reported [72–74], one representative approach is depicted in Fig. 3 [72]. Starting with pregnenolone (GANA-001), reduction of double bond with hydrogen catalyzed by Pd/C affords compound GANA-002. The subsequent oxidation reaction of GANA-002 with NaOCl and NaBr gives diketone GANA-003, followed by epoxidation reaction to provide epoxide GANA-004. Finally, GANA-004 undergoes NaI promoted ring-opening producing ganaxolone.

### Sodium phenylbutyrate/taurursodiol (Relyvrio)

Phenylbutyric acid, a fatty acid derivative of butyric acid produced by natural fermentation of colon bacteria, has many cellular and biological effects, such as easing inflammation, which is used to treat neurological or urea cycle disorders and inherited metabolic syndrome [75]. Sodium phenylbutyrate is a prodrug of phenylacetic acid that quickly metabolizes to its original style [76]. Then, phenylacetate binds to phenylacetyl-CoA, which is subjected to acetylation to give phenylacetylglutamine, which is ultimately excreted by the kidneys [77]. In Europe, taurursodiol, a taurine conjugate of ursodeoxycholic acid, is utilized for the prevention and treatment of gallstones due to its antiapoptotic and inhibitory effects on ER stress response [78]. Furthermore, taurursodiol has been also investigated in neurodegenerative and inflammatory metabolic diseases due to its array of molecular properties, such as anti-apoptotic effects [79, 80]. Taurursodiol effectively decreases the body cholesterol content and intake of dietary cholesterol by reducing intestinal absorption of cholesterol [81]. Relyvrio, an FDA-approved treatment for amyotrophic lateral sclerosis (ALS), is a blend of sodium phenylbutyrate and taurursodiol [82, 83]. ALS is so deadly that most patients have a life expectancy of only 3 to 5 years after onset of symptoms, and they typically die from respiratory failure, a progressive atrophy of the muscles used for respiration [84–87]. Relyvrio received early FDA approval due to the highly progressive nature and serious threat of ALS. However, Relyvrio prolong patients’ survival by slowing progression but not cure disease [88, 89].

The synthesis of taurursodiol was disclosed by Sandhill One, LLC in 2022 (Fig. 3) [90]. Condensations of 3-ketochol-4-enoic acid (TAUR-001) with 2,2-dimethyl-1,3-propanediol (TAUR-002) and MeOH in the presence of *p*-toluenesulfonic acid (TsOH), giving TAUR-003. Subsequent hydroxylation and oxidation with NHPI (*N*-hydroxyphthalimide) and pyridinium dichromate (PDC) form TAUR-004. Next, TAUR-004 is reduced with H_2_, followed by oxidation with pyridinium chlorochromate (PCC) to provide TAUR-005 over two steps. Treatment of compound TAUR-005 with HCOOH provides TAUR-006, in which the carbonyl is deprotected. One of the carbonyls of TAUR-006 then undergoes reduction, and the ester group is hydrolyzed at the same time, giving TAUR-007. After reduction of the other carbonyl, compound TAUR-008 reacts with taurine sodium salt (TAUR-009), giving taurursodiol.

## Dermatologic drugs

### Abrocitinib (Cibinqo)

On December 10, 2021, abrocitinib was initially approved by the European Commission to treat adult patients with atopic dermatitis (AD) [91, 92]. On January 14, 2022, abrocitinib received the FDA approval to treat patients with refractory moderate-to-severe AD who have limited or little response to other systemic drugs [93]. Abrocitinib potently and selectively inhibits JAK1 with an IC_50_ value of 29 nM, which is better than that of JAK2 (IC_50_ = 803 nM). Abrocitinib exerts anti-inflammatory effects by blocking pro-inflammatory cytokine signaling associated with atopic dermatitis [94]. It effectively decreases serum markers of atopic dermatitis inflammation in a dose-dependent manner, including interleukin-31 (IL-31), thymus and activation-regulated chemokine (TARC), and high-sensitivity C-reactive protein (hsCRP) [95]. Mean absolute lymphocyte counts increased during two weeks of treatment and returned to baseline after 9 months of treatment [93, 96, 97]. According to the Phase 3 results, the experimental group showed significant improvement in disease extent, severity, and skin clarity compared to the placebo group, and patients were able to rapidly relieve itching symptoms after two weeks treatment [94]. It is one of the first oral JAK inhibitors for AD in the United States, and previously received FDA breakthrough treatment and priority review qualifications [98].

**Fig. 4:**
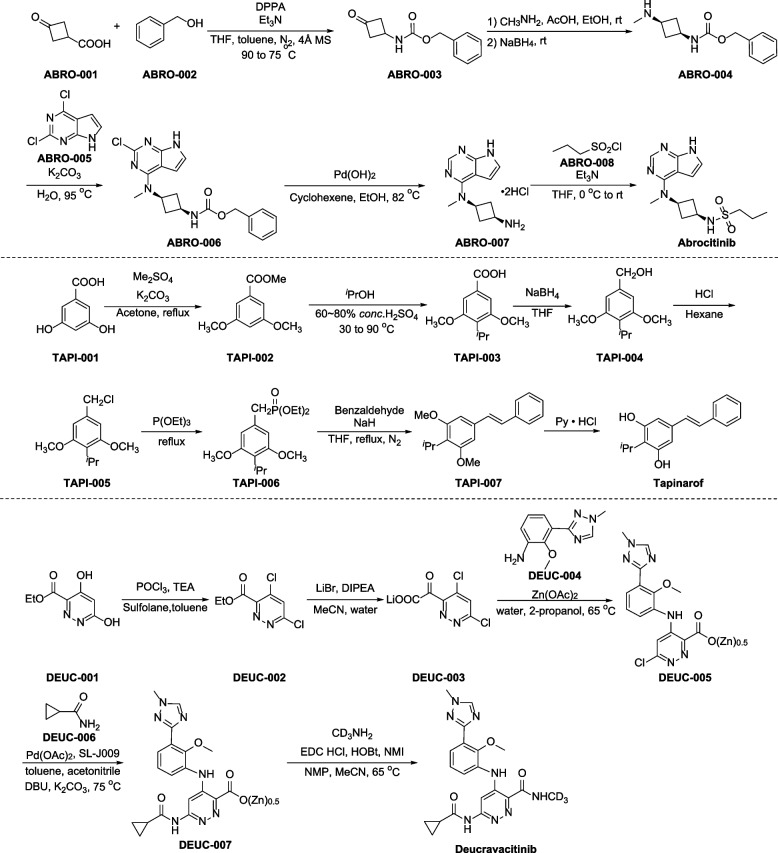
Synthesis of abrocitinib, tapinarof and deucravacitinib

Several synthetic methods of abrocitinib have been reported [95, 99–103], one representative synthetic route is described in Fig. 4 [104]. Curtius rearrangement and addition of 3-oxocyclobutane-1-carboxylic acid (ABRO-001) with phenylmethanol (ABRO-002) give ABRO-003 [105]. Next, treatment of compound ABRO-003 with monomethylamine in acetic acid, which then undergoes NaBH_4_-catalyzed reduction reaction to provide chiral compound ABRO-004. Treatment of ABRO-004 with pyrimidine ABRO-005 in the presence of K_2_CO_3_ provides the corresponding substitution product ABRO-006, followed by dechlorination and hydrolysis, giving salt ABRO-007. Finally, treatment of ABRO-007 with propane-1-sulfonyl chloride (ABRO-008) provides nucleophilic substitution product abrocitinib.

### Tapinarof (Vtama)

Tapinarof was approved by FDA on May 23, 2022, to treat plaque psoriasis [106, 107]. Tapiranof was originally identified as a metabolite (3, 5-dihydroxy-4-isopropyl styrenes) generated by Photorhabdus luminescens, a species of gram-negative bacilli living together with allogenic nematodes [108]. As a first-in-class agonist of aryl hydrocarbon receptor (AhR), Tapinarof exhibits excellent potency toward AhR (EC_50_ = 13 nM), which effectively regulates antioxidant activity and skin barrier protein expression, and inhibits inflammatory cytokines [109]. Tapinarof directly binds to AhR, thereby activating the AhR pathway. Tapinarof induces AhR nuclear translocation in immortalized keratinocytes (HaCaT) in a dose-dependent manner (EC_50_ = 0.16 nM). The anti-inflammatory effect of tapinarof may be due to Nrf2, a downstream effector of AhR, but not all AhR agonists can activate the pathway [110]. Therefore, the dual AhR/Nrf2 action of tapinarof may be responsible for psoriasis therapy [111].

The total synthesis of tapinarof is depicted in Fig. 4 [112]. Methylation of commercially available dimethyl sulfate with 3,5-dihydroxybenzoic acid (TAPI-001), followed by reaction with isopropyl alcohol in the presence of 60% ~ 80% concentrated sulfuric acid, giving TAPI-003. Subsequently reduction of carboxyl forms alcohol TAPI-004. Next, chlorination of TAPI-004 with hydrochloric acid provides TAPI-005, which then undergoes Wittig-Horner condensation to generate compound TAPI-006. TAPI-006 reacts with benzaldehyde in the presence of NaH and THF under nitrogen atmosphere, affording TAPI-007, which is subjected to demethylation catalyzed by pyridine hydrochloride to afford the target compound tapinarof.

### Deucravacitinib (Sotyktu)

On September 9, 2022, Deucravacitinib was approved by the FDA to treat moderate-to-severe plaque psoriasis [113]. Deucravacitinib, a member of the Janus kinase (JAK) family, is a highly potent allosteric inhibitor of tyrosine kinase 2 (TYK2) with an IC_50_ value of 1.0 nM [114]. It stabilizes an inhibitory interaction between catalytic and regulatory domains of the enzyme, which blocks the activation of Signal Transducers and Activators of Transcription (STATs) and TYK2in cell-based assays [115–117]. The precise mechanism by which inhibiting the TYK2 enzyme leads to effective treatment in patients with moderate-to-severe plaque psoriasis is still not fully understood. However, its mechanism is different from other Janus kinase inhibitors targeting the conserved active domain, thereby exerting its high selectivity toward TYK2 [118], which is expected to avoid various adverse effects caused by non-selective JAK inhibitors, such as kidney and liver dysfunction, and altered triglyceride and cholesterol level.

Preparation of deucravacitinib is outlined in Fig. 4 [119]. Chlorodehydration of 4,6-dihydroxypyridazine-3-carboxylate (DEUC-001) with phosphorus oxychloride affords the corresponding dichloride DEUC-002, which undergoes hydrolysis in the presence of lithium bromide and Hunig’s base in aqueous acetonitrile to yield the lithium carboxylate DEUC-003. Nucleophilic aromatic substitution with DEUC-004 takes place at C4 position of DEUC-003, in the presence of zinc acetate, leading to the formation of DEUC-005 as a zinc salt. Subsequent coupling with cyclopropanecarboxamide (DEUC-006) is catalyzed by palladium acetate and a Josiphos ligand to generate compound DEUC-007. Finally, DEUC-007 undergoes an amidation with methan-d_3_-amine hydrochloride in the presence of 1-ethyl-3-(3-dimethylaminopropyl)carbodiimide hydrochloride (EDC), 1-hydroxybenzotriazole (HOBt) and *N*-methylimidazole (NMI), affording deucravacitinib.

## Hematologic drugs

### Mitapivat (Pyrukynd)

Mitapivat is the first orally active pyruvate kinase allosteric activator, which was approved by FDA on February 17, 2022, to manage hemolytic anemia in individuals with pyruvate kinase (PK) deficiency [120–122]. Mitapivat activates PK through allosteric regulation, binding to a distinct allosteric site on the PKR tetramer separate from fructose bisphosphate FBP [123]. The red blood cell (RBC) form of PK is mutated in PK deficiency, resulting in shortened RBC lifespan, reduced adenosine triphosphate (ATP), and chronic hemolysis [124]. Mitapivat increases its affinity for its substrate and stabilizes phosphoenolpyruvate by binding to pyruvate kinase [120]. Mitapivat increases ATP production and erythrocyte pyruvate kinase activity (wild-type and mutant forms) but reduces levels of 2,3-DPG [123]. Mitapivat has also been investigated in other genetic disorders affecting red blood cells and causing hemolytic anemia, such as α-/β-thalassemia and sickle cell disease [123].

**Fig. 5:**
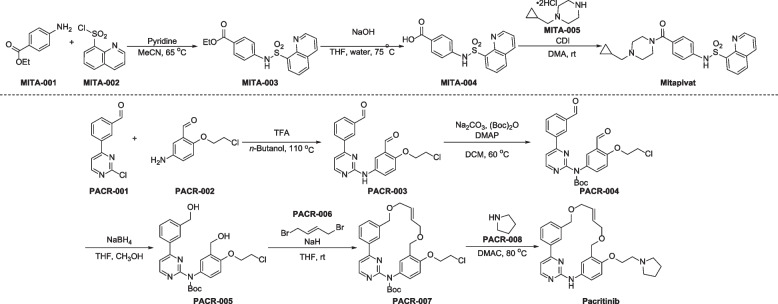
Synthesis of mitapivat and pacritinib

The preparation of mitapivat developed by Agios is shown in Fig. 5 [125, 126]. Starting with ethyl 4-aminobenzoate (MITA-001), a nucleophilic substitution reaction with quinoline-8-sulfonyl chloride (MITA-002), followed by NaOH promoted hydrolysis, giving MITA-004. Finally, the condensation of MITA-004 with 1-(cyclopropylmethyl)piperazine (MITA-005) in the presence of 1,1'-carbonyldiimidazole (CDI) gives the desired mitapivat.

### Pacritinib (Vonjo)

On February 28, 2022, the FDA granted accelerated approval to Pacritinib, a highly effective inhibitor of JAK2 and FMS-like tyrosine kinase 3 (FLT3), which was used to treat adult patients with low platelets who suffer from intermediate or high-risk primary or secondary myelofibrosis (MF) [127, 128]. Pacritinib potently inhibits wild-type JAK2 (IC_50_ = 23 nM), JAK2^V617F^ (IC_50_ = 19 nM), FLT3 (IC_50_ = 22 nM), and FLT3^D835Y^ (IC_50_ = 6 nM) [129], which benefits the signaling of many growth factors and cytokines associated with immune and hematopoiesis function. MF is closely related to dysregulated JAK2 signaling. Pacritinib carries significant selectivity for JAK2 over JAK3 and TYK2, and does not inhibit JAK1 at clinically relevant concentrations [130].

Pacritinib demonstrates a dose-dependent suppression of signal transducer and activator of transcription 5 (STAT5) phosphorylation in expanded erythroid progenitor cells obtained from healthy individuals [131]. A single 400 mg dose of pacritinib moderately inhibits STAT3 phosphorylation induced by interleukin 6 in the whole blood of healthy subjects [132, 133]. Pacritinib is administered orally twice daily and is contraindicated in patients with renal insufficiency and hepatic insufficiency. It offers an alternative for MF patients with severe thrombocytopenia, commonly carrying a pretty poor prognosis [134].

Although several synthetic routes to pacritinib have been reported [135, 136], an efficient approach has been disclosed in Fig. 5 [137]. Substitution of 3-(2-chloropyrimidin-4-yl)benzaldehyde (PACR-001) with 5-amino-2-(2-chloroethoxy)benzaldehyde (PACR-002), followed by *N*-Boc protection of compound PACR-003 with di-*tert*-butyl pyrocarbonate, generating the key intermediate PACR-004. Further NaBH_4_ promoted reduction of PACR-004 furnishes PACR-005. Treatment of PACR-005 with (*E*)-1,4-dibromobut-2-ene (PACR-006) in basic solution gives the ring-closing product PACR-007, which undergoes nucleophilic substitution reaction with pyrrolidine (PACR-008) to provide the desired pacritinib.

## Oncologic drugs

### Lutetium (^177^Lu) vipivotide tetraxetan (Pluvicto)

As a radioligand therapeutic agent, lutetium (^177^Lu) vipivotide tetraxetan consists of a radionuclide, lutetium Lu-177, conjugated to a prostate-specific membrane antigen (PSMA)-binding moiety, exercising cytotoxic effect on cancer cells [138, 139]. The β-negative emission of lutetium Lu-177 irradiates PSMA-expressing cells and surrounding cells, thereby inducing DNA damage and cell death. In a clinical trial, it was discovered that Lutetium (^177^Lu) vipivotide tetraxetan exhibited a significant correlation with an 80.4% decrease in serum PSA levels. Additionally, the median progression-free survival for these patients was determined to be 13.7 months [140]. Following other therapies, it was granted FDA approval on March 23, 2022, to treat metastatic castration-resistant prostate cancer with prostate-specific membrane antigen positivity [141]. This approval is based on the positive results of phase 3 clinical trial, which showed that the addition of Pluvicto reduced the risk of patient death by 38% compared to standard therapy and that Pluvicto also significantly reduced the risk of patients developing radiographic disease progression or death [142]. Furthermore, in patients with evaluable disease at baseline, the overall remission rate was 30% in the Pluvicto group, compared to 2% in the standard treatment control group [143]. In October 2022, the Committee for Medicinal Products for Human Use (CHMP) of the European Medicines Agency (EMA) issued a recommendation for the authorization of marketing for a therapeutic intervention targeting prostate cancer [144].

**Fig. 6:**
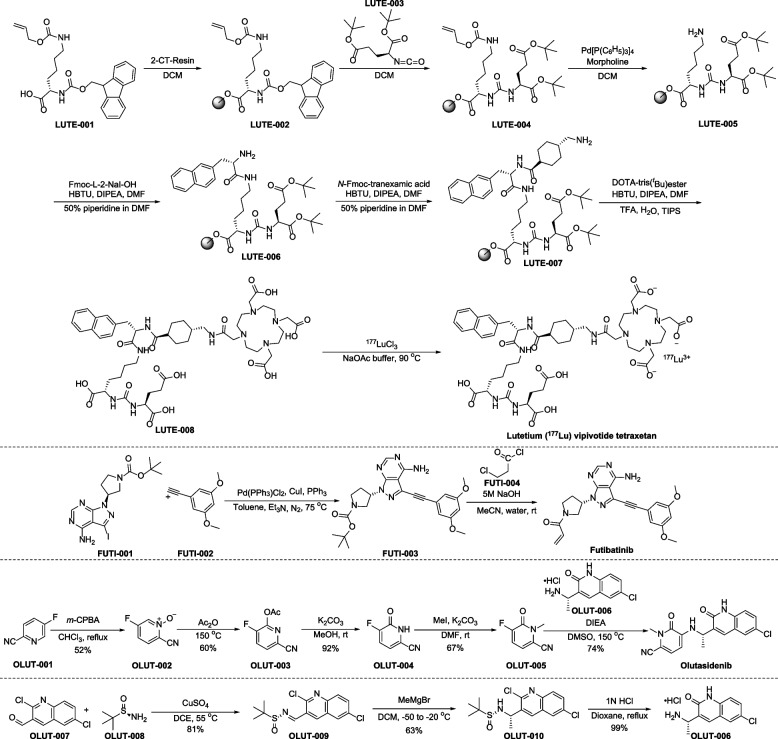
Synthesis of lutetium (^177^Lu) vipivotide tetraxetan, futibatinib and olutasidenib

Preparation of lutetium (^177^Lu) vipivotide tetraxetan is depicted in Fig. 6 [145, 146]. Starting from 2-chlotrotrityl chloride (2-CT) resin, installation of LUTE-001 is accomplished in the presence of DCM to furnish LUTE-002. From LUTE-002, condensation with isocyanic acid LUTE-003 provides the intermediate LUTE-004. Treatment of LUTE-004 with Pd[P(C_6_H_5_)_3_]_4_ and morpholine, followed by condensation with Fmoc-L-2-NaI-OH, forming LUTE-006. Following the same procedure, LUTE-007 is synthesized. Further condensation and radiolabelling with ^177^Lu give Lutetium (^177^Lu) vipivotide tetraxetan.

### Futibatinib (Lytgobi)

On September 30, 2022, the FDA granted approval to Futibatinib, a permanent inhibitor of Fibroblast Growth Factor receptor (FGFR), for the treatment of intrahepatic cholangiocarcinoma that contains FGFR2 gene fusions or other genetic rearrangements [147]. Futibatinib effectively inhibits FGFR 1–4 (IC_50_ = 3.9, 1.3, 1.6, and 8.3 nM), respectively, by forming a covalent bond with cysteine in the ATP-binding pocket of FGFR kinase domain [148–150]. In addition, Futibatinib exhibits similar inhibitory potency toward wild-type (WT) and mutant FGFR2 with IC_50_ values of 0.9 nM, 1–3 nM, 3.6 nM, and 2.4 nM against WT FGFR2, V5651, N550H, and E566G, respectively [148–150]. FGFR plays a crucial role in cell differentiation, survival, proliferation, and migration, and aberrant signaling pathways and genomic aberrations commonly occur in a variety of cancers since the survival and proliferation of malignant cells can be supported by FGFR signaling [148, 151]. Futibatinib efficiently inhibits the phosphorylation of FGFR and subsequent signaling pathways. This inhibition leads to a decrease in the survival of cancer cells harboring FGFR rearrangements, fusions, mutations, and amplifications in xenograft models derived from mice and rats [152]. In the end, futibatinib effectively decreases the survival of cancer cells with FGFR alterations, such as FGFR fusions or rearrangements, amplifications, and mutations [153].

Futibatinib is prepared as described in Fig. 6 [154]. Sonogashira coupling between FUTI-001 and l-ethynyl-3,5-dimethoxybenzene (FUTI-002) gives compound FUTI-003, followed by the treatment of FUTI-003 with 3-chloropropionyl chloride (FUTI-004), affording the product futibatinib.

### Olutasidenib (Rezlidhia)

On December 1, 2022, the FDA granted approval to olutasidenib, a highly effective inhibitor of isocitrate dehydrogenase-1 (IDH1), to treat relapsed or refractory acute myeloid leukemia (AML) in adults who possess a susceptible IDH1 mutation [155, 156]. Normally, IDH1 catalyzes mediated the conversion of isocitrate to α-ketoglutarate (α-KG) through oxidative decarboxylation reaction [157]. However, IDH1 mutations are commonly observed in the catalytic sites of arginine in various cancers, such as AML, and stimulate the transfer of α-KG to 2-hydroxyglutarate (2-HG) [158]. This leads to 2-HG increase, which is closely related to the inhibition of α-KG-dependent mechanisms, such as collagen synthesis, epigenetic regulation, and cell signaling. Olutasidenib effectively decreases 2-HG levels by selectively targeting mutant IDH1, leading to the restoration of normal cell differentiation and offering therapeutic advantages in IDH1 mutant strains. Additionally, olutasidenib is currently investigated to treat myelodysplastic syndromes (MDS), as well as solid tumors and gliomas (NCT03684811) [159–161].

The synthetic route of olutasidenib as described in the publication is shown in Fig. 6 [162]. *N*-oxidation of commercially available 5-fluoropicolinonitrile (OLUT-001) followed by reflux of the *N*-oxide (OLUT-002) in acetic anhydride give the acetate OLUT-003. Base-mediated hydrolyzation and tautomerism of OLUT-003, followed by *N*-methylation with methyl iodide provide *N*-methylated compound OLUT-005. Finally, the condensation of OLUT-005 with the amine OLUT-006 affords olutasidenib.

**Fig. 7:**
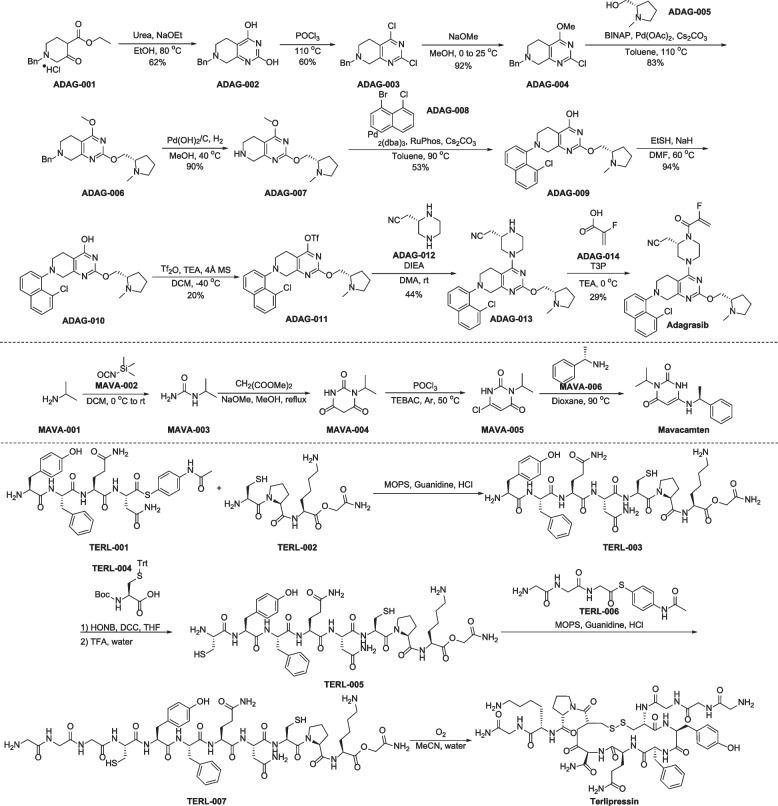
Synthesis of adagrasib, mavacamten and terlipressin

Of note, the preparation of the amine OLUT-006 arises from commercially available quinoline aldehyde OLUT-007, which is first condensed with (*R*)-*tert*-butanesulfinamide (OLUT-008) to obtain the chiral (*R*)-*N*-*tert*-butanesulfinimine (OLUT-009) in 81% yield (Fig. 7). Next, an addition reaction of OLUT-009 and methylmagnesium bromide in dichloromethane is employed to yield the intermediate OLUT-010 as the major diastereoisomer (98:2 dr). Removal of the sulfonyl group using hydrochloric acid provides the intermediate OLUT-006 in quantitative yield.

### Adagrasib (Krazati)

Adagrasib, an orally bioavailable inhibitor of KRAS, was developed by Mirati. On December 12, 2022, Therapeutics obtained accelerated FDA approval to treat KRAS G12C-mutated locally advanced or metastatic non-small cell lung cancer in adults who have undergone at least one prior systemic therapy [163–165]. Normally, activation of KRAS through binding to guanosine triphosphate (GTP) stimulates the activation of intracellular signal transduction and MAP kinase pathway. Hydrolyzation of GTP gives guanosine diphosphate (GDP) and KRAS restores the inactive state [166]. Cysteine substitution of Gly12 in KRAS (KRASG12C) damages GTP hydrolysis and keeps KRAS in active form, which results in uncontrolled cell growth and proliferation as well as malignant transformation [167]. Adagrasib covalently and selectively binds to KRASG12C and locks it in the inactive state, thus exerting anti-tumor activity by inhibiting tumor cell viability and growth [168].

The synthesis of adagrasib begins by condensing ethyl 1-benzyl-3-oxopiperidine-4-carboxylate (ADAG-001) with urea. This reaction forms ADAG-002, which is a bicyclic diol. ADAG-002 is then chlorinated using POCl_3_ to produce pyrimidine (ADAG-003) (Fig. 7) [169]. Next, treatment of intermediate ADAG-003 with sodium methoxide in methanol, followed by Buchwald coupling with (*S*)-(1-methylpyrrolidin-2-yl)methanol (ADAG-005), gives compound ADAG-006. *N*-debenzylation of ADAG-006 in the presence of hydrogen catalyzed by Pd(OH)/C affords the amine ADAG-007, which is treated with 1-bromo-8-chloronaphthalene (ADAG-008) under Buchwald-Hartwig amination conditions to give the *N*-arylated product ADAG-009. Demethylation and subsequent triflate formation provide ADAG-011, which reacts with (*S*)-2-(piperazin-2-yl)acetonitrile (ADAG-012) affording the advanced intermediate ADAG-013. It then undergoes ammonolysis with 2-fluoroacrylic acid using T3P as the coupling reagent to obtain adagrasib [170].

## Other drugs

### Mavacamten (Camzyos)

Mavacamten was approved by US FDA in 2022, which is used to treat adult patients with obstructive hypertrophic cardiomyopathy (oHCM) who are experiencing symptoms and fall into New York Heart Association (NYHA) class II-III [171, 172]. Mavacamten is a medication that can be taken by mouth. It acts as an inhibitor of cardiac myosin, which is a protein involved in muscle contraction in the heart. This medication works by regulating the number of myosin heads that can enter the "on actin" states, effectively reducing the likelihood of generating force during systole (contraction) and residual force during diastole (relaxation) across the bridge. Additionally, its inhibitory effects are reversible, meaning that its action can be reversed if necessary [173]. HCM is characterized by the presence of excessive formation of myosin actin bridges and dysregulation of the hyperrelaxation state [174]. Mavacamten transforms the total amount of myosin into an energy-saving, absorbable, ultra-relaxed state [175]. Inhibition of myosin with Mavacamten reduced dynamic left vein obstruction and improved cardiac filling pressure in patients with HCM. It has been reported that the IC_50_ values of Mavacamten in the bovine, human, and rabbit system are 490 nM, 711 nM, and 2140 nM, respectively, indicating a 4-fold selectivity for myocardial myosin [176–178]. Mavakamten is found to reduce contractility through decreasing the activity of adenosine triphosphatase in the heavy chain of myocardial myosin [179]. Long-term administration results in the inhibition of the development of myocardial cell disorders, myocardial hypertrophy, and myocardial fibrosis, and attenuates the gene expression of profibrotic and hypertrophic in mice featuring heterozygous human mutations in the chain of myosin heavy [176].

A convenient synthetic method of mavacamten was disclosed in 2014 (Fig. 7) [180]. Addition of commercially available isopropylamine (MAVA-001) with trimethylsilyl isocyanate (MAVA-002), followed by the annulation reaction with dimethyl malonate in the presence of sodium methoxide and methanol, giving MAVA-003. Subsequently, chlorination forms compound MAVA-005. Finally, the coupling of MAVA-005 with (*S*)-α-methylbenzylamine (MAVA-006) in dioxane under 90 °C provides desired mavacamten.

### Terlipressin (Terlivaz)

Terlipressin, a medication aimed at enhancing kidney function in adult patients with hepatorenal syndrome experiencing a rapid decline in kidney function, obtained FDA approval on September 14, 2022 [181, 182]. Terlipressin, an analog of vasopressin, is an endogenous neurohormone that acts as a vasoconstrictor [183–188]. As a prodrug of lysine-vasopressin, terlipressin itself is pharmacologically active, characterized by a longer half-life and higher selectivity for V1 receptor than vasopressin, which controls acute variceal bleeding, and reduces the splanchnic blood flow and portal pressure [189]. These favorable pharmacokinetic and molecular properties of terlipressin confer several advantages, such as convenience in patients with limited intravenous access and prevention of rebound hypotension upon discontinuation [188, 190].

Preparation of terlipressin is described in Fig. 7 [191]. Condensation of thioester TERL-001 and *N*-terminal cysteine TERL-002 furnishes TERL-003 in the presence of 3-(*N*-morpholino)propanesulfonic (MOPS) and catalytic guanidine. Subsequent condensation of TERL-003 with Boc-Cys(trt)-OH TERL-004 and *N*-Boc deprotection furnish TERL-005, further guanidine-catalyzed condensation with TERL-006 gives TERL-007. Oxidation of TERL-007 in MeCN/H_2_O gives terlipressin.

### Gadopiclenol (Elucirem)

Gadopiclenol, a gadolinium-based contrast agent (GBCA), was developed by Guerbet. On September 21, 2022, after undergoing a priority review, the FDA granted its approval to the product. Its primary objective is to identify and display abnormal vascularity lesions in the body and the central nervous system, in combination with magnetic resonance imaging (MRI). In 2006, linear GBCA use was linked to nephrogenic systemic fibrosis (NSF) [192–194], a rare disease characterized by hardening and thickening of the subcutaneous and skin tissue, which has not been detected in macrocyclic GBCAs, such as gadopiclenol [195]. It should be noted that in NSF patients with impaired drug clearance, a black box warning was given to gadopiclenol to remind an increased risk. Gadopiclenol carries two water molecule exchange sites for increased relaxation and contrast [195]. Compared with other non-specific GBCAs, gadopiclenol dose is only half of the conventional gadolinium dose, alleviating practitioners' concerns about radiation exposure [196]. Variations in radiofrequency signal strength allow visualization of normal and pathological tissue during MRI, which is characterized by the differences in longitudinal relaxation times (T1) or spin–lattice, proton density, or in transverse relaxation times or spin–spin (T2). The T1 and T2 relaxation times can be shortened by Gadopiclenol, resulting in the visualization of target tissues during MRI [197]. The extent of a contrast agent affecting the tissue water relaxation rate (1/T1 or 1/T2) is expressed in terms of the relaxation rate (r1 or r2). The high r1 relaxation rate and kinetic stability of gadopiclenol allow it to be used at lower doses than traditional extracellular GBCAs. What deserves special vigilance is that acute kidney injury and hypersensitivity reactions may also occur with gadopiclenol [198].

**Fig. 8:**
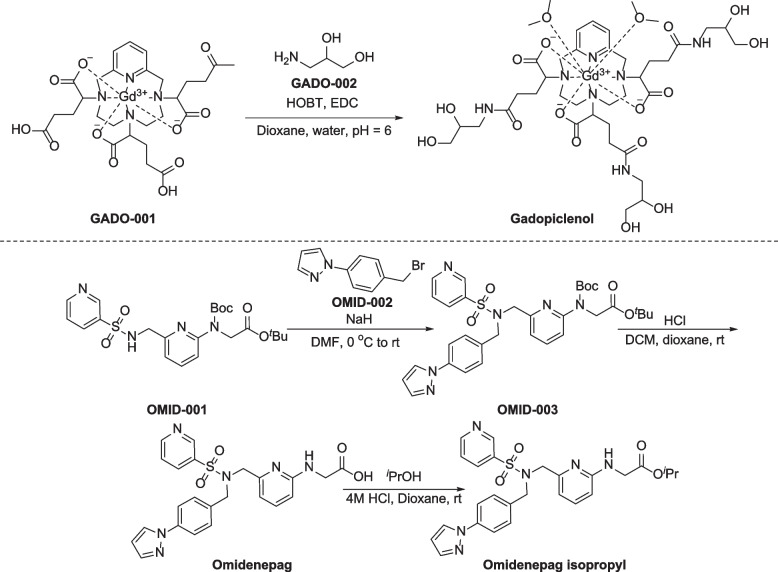
Synthesis of gadopiclenol and omidenepag isopropyl

The reported synthesis of gadopiclenol was reported by Marc, in 2007 (Fig. 8) [199]. Treatment of compound GADO-001 with 3-aminopropyl-1,2-diol (GADO-002) in the presence of HOBt and EDC gives gadopiclenol.

### Omidenepag isopropyl (Omlonti)

Omidenepag isopropyl, approved in Japan in 2018 as a potent agonist of EP2 receptor to treat ocular hypertension and glaucoma [200], was then approved by FDA on September 22, 2022, to lower high intraocular pressure in individuals diagnosed with ocular hypertension or open-angle glaucoma. Omidenepag isopropyl is rapidly metabolized to its active metabolite omidenepag, which binds strongly to prostaglandin E2 (EP2) receptor (Ki = 3.6 nM) [201]. Furthermore, Omidenepag is highly agonistic at the EP2 receptor (EC_50_ = 8.3 nM) but has little effect on other receptors such as prostaglandin E1 (EP1) or F receptors (FP) [200, 202, 203]. Unlike omidenepag, omidenepag isopropyl has little or no affinity for prostaglandin receptors [200, 203]. EP2 receptor exists in different kinds of ocular tissues related to aqueous humor dynamics, such as ciliary muscle (CM) and trabecular meshwork (TM) [204]. Stimulation of EP2 receptors may result in increased intracellular cyclic adenosine monophosphate (cAMP), leading to relaxation of CM and TM tissues [205]. Omidenepag has shown comparable efficacy in lowering IOP to latanoprost, a prostaglandin FP receptor agonist, which is a first-line treatment for primary ocular hypertension and open-angle glaucoma [206].

Preparation of omidenepag isopropyl is depicted in Fig. 8 [202]. Treatment of OMID-001 with 1-(4-(bromomethyl)phenyl)-1*H*-pyrazole (OMID-002) provides the corresponding coupling product OMID-003, followed by HCl-promoted hydrolysis and *N*-Boc deprotection, producing omidenepag. Esterification of omidenepag with isopropyl alcohol in dioxane yields omidenepag isopropyl.

## Conclusion and prospect

In conclusion, in 2022, FDA approved 37 new drugs, including 20 NCEs, 7 monoclonal antibodies (mAbs), 3 bispecific antibodies (BsAbs), 2 enzymes, 1 fusion protein, 1 synthetic polypeptide, 1 small interfering RNA (siRNA), 1 toxin, 1contrast agent and a colony stimulating factor (CSF). In the context of the COVID-19 pandemic, FDA is still committed to supporting the development of rare disease drugs. The field of anti-tumor is still a hot area for innovative drug development. These approved new drugs will benefit patients suffering from orphans, cancer, nervous system diseases, infectious diseases, and many other diseases.

In this review, we summarized the clinic application and synthetic routes of the 19 NCEs of new drugs approved by the FDA. The pharmacophore library will be enriched and new drug discovery will be benefited by the presence of privileged scaffolds in these drug molecules. For example, me-better drug omidenepag isopropyl is developed based on the molecular structure of CP-533,536, which was reported by previous researchers and optimized through the structural modification of PGE2 [202]. The AIDS prevention drug lenacapavir is developed based on the structure of PF-3450074. In order to block the unstable metabolic site, researchers focused on introducing electron-withdrawing groups (halogens and sulfonyl groups) and metabolically stable rigid ring systems (cyclopropane and pyrazole) through optimization [207]. Both deucravacitinib and olutasidenib were first screened through high-throughput screening to obtain lead compounds, and their solubility was improved through SBDD. Adagrasib has increased its in vitro stability in whole blood (WB) by introducing a fluorine atom at the 2-position of acrylamide [208].

In addition, we discovered that metal-catalyzed coupling reactions were utilized in the synthesis of pharmaceuticals (such as omidenepag, futibatinib, oteseconazole, etc.). Chiral resolution and asymmetric synthesis were employed for obtaining the single enantiomer of the chiral drugs (such as taurursodiol, ganaxolone, and oteseconazole, etc.). Other traditional organic synthetic strategies, such as substitution reaction, hydrolysis reaction, Curtius rearrangement, and addition reaction were still used for the synthesis of these new drug molecules approved by the FDA. The special skeletons in the drug molecules enrich the effective pharmacophores, which will help to design new drugs.

### Supplementary Information

**Additional file 1.**

## Data Availability

Not applicable.

## References

[1] Mullard A (2023). 2022 FDA approvals. Nat Rev Drug Discov.

[2] Food and Drug Administration. Novel Drug Approvals for 2022. https://www.fda.gov/drugs/new-drugs-fda-cders-new-molecular-entities-and-new-therapeutic-biological-products/novel-drug-approvals-2022. Accessed Dec 31, 2022.

[3] Al-Madhagi HA (2023). FDA-approved drugs in 2022: A brief outline. Saudi Pharm J.

[4] Gabbard AP, Booth GS (2020). Cold agglutinin disease. Clin Hematol Int.

[5] Dhillon S (2022). Sutimlimab: First approval. Drugs.

[6] Tefferi A (2003). The forgotten myeloproliferative disorder: Myeloid metaplasia. Oncologist.

[7] De SK (2023). First approval of pacritinib as a selective janus associated kinase-2 inhibitor for the treatment of patients with myelofibrosis. Anticancer Agents Med Chem.

[8] Hawkins PN, Ando Y, Dispenzeri A, Gonzalez-Duarte A, Adams D, Suhr OB (2015). Evolving landscape in the management of transthyretin amyloidosis. Ann Med.

[9] Keam SJ (2022). Vutrisiran: First approval. Drugs.

[10] Ding HX, Leverett CA, Kyne RE, Liu KK, Fink SJ, Flick AC (2015). Synthetic approaches to the 2013 new drugs. Bioorg Med Chem.

[11] Flick AC, Ding HX, Leverett CA, Kyne RE, Liu KK, Fink SJ (2016). Synthetic approaches to the 2014 new drugs. Bioorg Med Chem.

[12] Flick AC, Ding HX, Leverett CA, Kyne RE, Liu KK, Fink SJ (2017). Synthetic approaches to the new drugs approved during 2015. J Med Chem.

[13] Flick AC, Ding HX, Leverett CA, Fink SJ, O'Donnell CJ (2018). Synthetic approaches to new drugs approved during 2016. J Med Chem.

[14] Flick AC, Leverett CA, Ding HX, McInturff E, Fink SJ, Helal CJ (2019). Synthetic approaches to the new drugs approved during 2017. J Med Chem.

[15] Flick AC, Leverett CA, Ding HX, McInturff E, Fink SJ, Helal CJ (2020). Synthetic approaches to new drugs approved during 2018. J Med Chem.

[16] Yuan S, Yu B, Liu HM (2020). New drug approvals for 2019: Synthesis and clinical applications. Eur J Med Chem.

[17] Flick AC, Leverett CA, Ding HX, McInturff E, Fink SJ, Mahapatra S (2021). Synthetic approaches to the new drugs approved during 2019. J Med Chem.

[18] Yuan S, Luo YQ, Zuo JH, Liu H, Li F, Yu B (2021). New drug approvals for 2020: Synthesis and clinical applications. Eur J Med Chem.

[19] Flick AC, Leverett CA, Ding HX, McInturff EL, Fink SJ, Mahapatra S (2022). Synthetic approaches to the new drugs approved during 2020. J Med Chem.

[20] Yuan S, Wang DS, Liu H, Zhang SN, Yang WG, Lv M (2023). New drug approvals for 2021: Synthesis and clinical applications. Eur J Med Chem.

[21] Hoy SM (2022). Oteseconazole: First approval. Drugs.

[22] Sobel JD (2016). Recurrent vulvovaginal candidiasis. Am J Obstet Gynecol.

[23] Garvey E, Hoekstra W, Moore W, Schotzinger R, Long L, Ghannoum M (2015). VT-1161 dosed once daily or once weekly exhibits potent efficacy in treatment of dermatophytosis in a guinea pig model. Antimicrob Agents Ch.

[24] Warrilow A, Hull C, Parker J, Garvey E, Hoekstra W, Moore W (2014). The clinical candidate VT-1161 is a highly potent inhibitor of Candida albicans CYP51 but fails to bind the human enzyme. Antimicrob Agents Ch.

[25] De SK (2023). Oteseconazole: First approved orally bioavailable and selective CYP51 inhibitor for the treatment of patients with recurrent vulvovaginal candidiasis. Curr Med Chem.

[26] Sobel JD, Nyirjesy P (2021). Oteseconazole: An advance in treatment of recurrent vulvovaginal candidiasis. Future Microbiol.

[27] Chang YL, Yu SJ, Heitman J, Wellington M, Chen YL (2017). New facets of antifungal therapy. Virulence.

[28] Sun G, Thai SF, Lambert GR, Wolf DC, Tully DB, Goetz AK (2006). Fluconazole-induced hepatic cytochrome P450 gene expression and enzymatic activities in rats and mice. Toxicol Lett.

[29] Mast N, Zheng W, Stout CD, Pikuleva IA (2013). Antifungal azoles: Structural insights into undesired tight binding to cholesterol-metabolizing CYP46A1. Mol Pharmacol.

[30] de Oliveira HC, Bezerra BT, Rodrigues ML (2023). Antifungal development and the urgency of minimizing the impact of fungal diseases on public health. ACS Bio Med Chem Au.

[31] Sobel JD. Candida vulvovaginitis: Treatment. https://medilib.ir/uptodate/show/115170. Accessed Dec 20, 2022.

[32] Martens MG, Maximos B, Degenhardt T, Person K, Curelop S, Ghannoum M (2022). Phase 3 study evaluating the safety and efficacy of oteseconazole in the treatment of recurrent vulvovaginal candidiasis and acute vulvovaginal candidiasis infections. Am J Obstet Gynecol.

[33] Hoekstra WJ, Yates CM, Behnke M, Alimardanov A, David SA, Fry DF (2015). Preparation of an antifungal tetrazole compound.

[34] Garnock-Jones KP (2015). Vonoprazan: First global approval. Drugs.

[35] Suerbaum S, Michetti P (2002). *Helicobacter pylori* infection. N Engl J Med.

[36] Suzuki S, Kusano C, Horii T, Ichijima R, Ikehara H (2022). The ideal *Helicobacter pylori* treatment for the present and the future. Digestion.

[37] Echizen H (2016). The first-in-class potassium-competitive acid blocker, vonoprazan fumarate: Pharmacokinetic and pharmacodynamic considerations. Clin Pharmacokinet.

[38] Sugano K (2018). Vonoprazan fumarate, a novel potassium-competitive acid blocker, in the management of gastroesophageal reflux disease: Safety and clinical evidence to date. Therap Adv Gastroenterol.

[39] Vandecruys P, Baldewijns S, Sillen M, Van Genechten W, Van Dijck P. Oteseconazole: a long-awaited diversification of the antifungal arsenal to manage recurrent vulvovaginal candidiasis (RVVC). Expert Rev Anti Infect Ther. 2023;21(8):799–812. 10.1080/14787210.2023.2233696. 10.1080/14787210.2023.2233696 37449774

[40] Chey WD, Mégraud F, Laine L, López LJ, Hunt B, Smith N (2021). S1382 vonoprazan dual and triple therapy for *Helicobacter pylori* eradication. Am J Gastroenterol.

[41] Roque-Borda CA, Da Silva PB, Rodrigues MC, Di Filippo LD, Duarte JL, Chorilli M (2022). Pharmaceutical nanotechnology: Antimicrobial peptides as potential new drugs against WHO list of critical, high, and medium priority bacteria. Eur J Med Chem.

[42] Kiyotoki S, Nishikawa J, Sakaida I (2020). Efficacy of vonoprazan for *Helicobacter pylori* eradication. Intern Med.

[43] Kakiuchi T, Mizoe A, Yamamoto K, Imamura I, Hashiguchi K, Kawakubo H (2020). Effect of probiotics during vonoprazan-containing triple therapy on gut microbiota in *Helicobacter pylori* infection: A randomized controlled trial. Helicobacter.

[44] Kajino M, Hasuoka A, Tarui N, Takagi T (2006). Preparation of pyrrole derivatives as proton pump inhibitors.

[45] Lu X, Zhang Y, Huo L, Li Z, Zhao Q, Zhou J (2015). A kind of preparation method of vonoprazan fumarate.

[46] Ikemoto T, Mizufune H, Nagata T, Sera M, Fukuda N, Yamasaki T (2010). Process for the preparation of pyrrole compound.

[47] Geng F, Liu Y, Liu X (2015). Method for preparing vonoprazan fumarate.

[48] Margot NA, Naik V, VanderVeen L, Anoshchenko O, Singh R, Dvory-Sobol H (2022). Resistance analyses in highly treatment-experienced people with human immunodeficiency virus (HIV) treated with the novel capsid HIV inhibitor lenacapavir. J Infect Dis.

[49] Dvory-Sobol H, Shaik N, Callebaut C, Rhee MS (2022). Lenacapavir: A first-in-class HIV-1 capsid inhibitor. Curr Opin HIV AIDS.

[50] Zhuang S, Torbett BE (2021). Interactions of HIV-1 capsid with host factors and their implications for developing novel therapeutics. Viruses.

[51] Margot N, Ram R, Rhee M, Callebaut C (2021). Absence of lenacapavir (GS-6207) phenotypic resistance in HIV gag cleavage site mutants and in isolates with resistance to existing drug classes. Antimicrob Agents Ch.

[52] Bester SM, Wei G, Zhao H, Adu-Ampratwum D, Iqbal N, Courouble VV (2020). Structural and mechanistic bases for a potent HIV-1 capsid inhibitor. Science.

[53] Nka AD, Bouba Y, Teto G, Semengue ENJ, Takou DK, Ngueko AMK (2022). Evaluation of HIV-1 capsid genetic variability and lenacapavir (GS-6207) drug resistance-associated mutations according to viral clades among drug-naive individuals. J Antimicrob Chemother.

[54] Bester SM, Adu-Ampratwum D, Annamalai AS, Wei G, Briganti L, Murphy BC (2022). Structural and mechanistic bases of viral resistance to HIV-1 capsid inhibitor lenacapavir. mBio.

[55] Allan KM, Batten AL, Brizgys G, Dhar S, Doxsee IJ, Goldberg A (2019). Methods and intermediates for preparation of antiretroviral pyridine derivative useful for treatment of HIV-1 infections.

[56] Markham A (2022). Daridorexant: First approval. Drugs.

[57] Morin AK, Jarvis CI, Lynch AM (2007). Therapeutic options for sleep-maintenance and sleep-onset insomnia. Pharmacotherapy.

[58] Roecker AJ, Cox CD, Coleman PJ (2016). Orexin receptor antagonists: New therapeutic agents for the treatment of insomnia. J Med Chem.

[59] Onge ES, Phillips B, Rowe C (2022). Daridorexant: a new dual orexin receptor antagonist for insomnia. J Pharm Technol.

[60] Roch C, Bergamini G, Steiner MA, Clozel M (2021). Nonclinical pharmacology of daridorexant: A new dual orexin receptor antagonist for the treatment of insomnia. Psychopharmacology.

[61] Herring WJ, Roth T, Krystal AD, Michelson D (2019). Orexin receptor antagonists for the treatment of insomnia and potential treatment of other neuropsychiatric indications. J Sleep Res.

[62] Boss C, Brotschi C, Gude M, Heidmann B, Sifferlen T, Von Raumer M (2015). Crystalline salt form of (S)-(2-(6-chloro-7-methyl-1 h-benzo[d]imidazol-2-yl)-2-methylpyrrolidin-1-yl)(5-methoxy-2-(2H-1,2,3-triazol-2-yl)phenyl)methanone as orexin receptor antagonist.

[63] Nohria V, Giller E (2007). Ganaxolone. Neurotherapeutics.

[64] Olson HE, Demarest ST, Pestana-Knight EM, Swanson LC, Iqbal S, Lal D (2019). Cyclin-dependent kinase-like 5 deficiency disorder: Clinical review. Pediatr Neurol.

[65] Vaitkevicius H, Ramsay RE, Swisher CB, Husain AM, Aimetti A, Gasior M (2022). Intravenous ganaxolone for the treatment of refractory status epilepticus: Results from an open-label, dose-finding, phase 2 trial. Epilepsia.

[66] Monaghan EP, Navalta LA, Shum L, Ashbrook DW, Lee DA (1997). Initial human experience with ganaxolone, a neuroactive steroid with antiepileptic activity. Epilepsia.

[67] Reddy DS, Woodward R (2004). Ganaxolone: A prospective overview. Drugs Future.

[68] Belelli D, Lambert JJ (2005). Neurosteroids: Endogenous regulators of the GABAA receptor. Nat Rev Neurosci.

[69] Dichtel LE, Nyer M, Dording C, Fisher LB, Cusin C, Shapero BG (2020). Effects of open-label, adjunctive ganaxolone on persistent depression despite adequate antidepressant treatment in postmenopausal women: A pilot study. J Clin Psychiatry.

[70] Wilkinson ST, Sanacora G (2019). A new generation of antidepressants: An update on the pharmaceutical pipeline for novel and rapid-acting therapeutics in mood disorders based on glutamate/GABA neurotransmitter systems. Drug Discov Today.

[71] Gonda X, Dome P, Neill JC, Tarazi FI (2023). Novel antidepressant drugs: Beyond monoamine targets. CNS Spectr.

[72] Reddy DS (2019). Preparation of Neurosteroid compounds and use in treating central nervous system disorders.

[73] Martinez Botella G, Salituro FG, Robichaud AJ, Harrison BL (2016). Preparation of neuroactive steroids, compositions and methods for treating CNS disorders.

[74] He M-h, Liao Q-j (2005). Synthesis of ganaxalone. Chin J New Drug.

[75] Kusaczuk M, Bartoszewicz M, Cechowska-Pasko M (2015). Phenylbutyric Acid: Simple structure-multiple effects. Curr Pharm Des.

[76] Kasumov T, Brunengraber LL, Comte B, Puchowicz MA, Jobbins K, Thomas K (2004). New secondary metabolites of phenylbutyrate in humans and rats. Drug Metab Dispos.

[77] Koetsier MJ, Jekel PA, van den Berg MA, Bovenberg RA, Janssen DB (2009). Characterization of a phenylacetate-CoA ligase from Penicillium chrysogenum. Biochem J.

[78] Albanese A, Ludolph AC, McDermott CJ, Corcia P, Van Damme P, Van den Berg LH (2022). Tauroursodeoxycholic acid in patients with amyotrophic lateral sclerosis: The TUDCA-ALS trial protocol. Front Neurol.

[79] Vang S, Longley K, Steer CJ, Low WC (2014). The unexpected uses of urso-and tauroursodeoxycholic acid in the treatment of non-liver diseases. Glob Adv Health Med.

[80] Ahn TK, Kim KT, Joshi HP, Park KH, Kyung JW, Choi UY (2020). Therapeutic potential of tauroursodeoxycholic acid for the treatment of osteoporosis. Int J Mol Sci.

[81] Lu Q, Jiang Z, Wang Q, Hu H, Zhao G (2021). The effect of tauroursodeoxycholic acid (TUDCA) and gut microbiota on murine gallbladder stone formation. Ann Hepato.

[82] Heo YA (2022). Sodium phenylbutyrate and ursodoxicoltaurine: First approval. CNS Drugs.

[83] Mullard A (2022). Amylyx's ALS therapy secures FDA approval, as regulatory flexibility trumps underwhelming data. Nat Rev Drug Discov.

[84] Morris J (2015). Amyotrophic lateral sclerosis (ALS) and related motor neuron siseases: An overview. Neurodiagn J.

[85] Hardiman O, Al-Chalabi A, Chio A, Corr EM, Logroscino G, Robberecht W (2017). Amyotrophic lateral sclerosis. Nat Rev Dis Primers.

[86] Feldman EL, Goutman SA, Petri S, Mazzini L, Savelieff MG, Shaw PJ (2022). Amyotrophic lateral sclerosis. Lancet.

[87] Meyer T (2021). Amyotrophic lateral sclerosis (ALS)-diagnosis, course of disease and treatment options. Dtsch Med Wochenschr.

[88] Paganoni S, Macklin EA, Hendrix S, Berry JD, Elliott MA, Maiser S (2020). Trial of sodium phenylbutyrate-taurursodiol for amyotrophic lateral sclerosis. New Engl J Med.

[89] Martinez-Gonzalez L, Martinez A (2023). Emerging clinical investigational drugs for the treatment of amyotrophic lateral sclerosis. Expert Opin Investig Drugs.

[90] Reid JG, Reddy JP, Paul BJ, Hossain SS (2022). Preparation of cholic acid derivatives.

[91] Niculet E, Bobeica C, Stefanopol IA, Pelin AM, Nechifor A, Onisor C (2022). Once-daily abrocitinib for the treatment of moderate-to-severe atopic dermatitis in adults and Adolescents Aged 12 Years and Over: A short review of current clinical perspectives. Ther Clin Risk Manag.

[92] Leung DY, Boguniewicz M, Howell MD, Nomura I, Hamid QA (2004). New insights into atopic dermatitis. J Clin Invest.

[93] Perche PO, Cook MK, Feldman SR (2023). Abrocitinib: A new FDA-approved drug for moderate-to-severe atopic dermatitis. Ann Pharmacother.

[94] Crowley EL, Nezamololama N, Papp K, Gooderham MJ (2020). Abrocitinib for the treatment of atopic dermatitis. Expert Rev Clin Immu.

[95] De SK (2023). Abrocitinib: First globally approved selective janus kinase-1 inhibitor for the treatment of atopic dermatitis. CurrMedChem.

[96] Simpson EL, Silverberg JI, Nosbaum A, Winthrop KL, Guttman-Yassky E, Hoffmeister KM (2021). Integrated safety analysis of abrocitinib for the treatment of moderate-to-severe atopic dermatitis from the phase II and phase III clinical trial program. Am J Clin Dermatol.

[97] Iznardo H, Roé E, Serra-Baldrich E, Puig L (2023). Efficacy and safety of JAK1 inhibitor abrocitinib in atopic dermatitis. Pharmaceutics.

[98] Mikhaylov D, Ungar B, Renert-Yuval Y, Guttman-Yassky E (2023). Oral JAK inhibitors for atopic dermatitis. Ann Allergy Asthma Immunol.

[99] Coffman KJ, Duerr JM, Kaila N, Parikh MD, Reese MR, Samad T (2016). Preparation of pyrrolo[2,3-d]pyrimidine derivatives and their use as Janus kinase inhibitors.

[100] Kumar R, Karmilowicz MJ, Burke D, Burns MP, Clark LA, Connor CG (2021). Biocatalytic reductive amination from discovery to commercial manufacturing applied to abrocitinib JAK1 inhibitor. Nat Catal.

[101] Kocienski P (2021). Synthesis of abrocitinib Synfacts.

[102] Vazquez ML, Kaila N, Strohbach JW, Trzupek JD, Brown MF, Flanagan ME (2018). Identification of N-{cis-3-[methyl(7H-pyrrolo[2,3-d]pyrimidin-4-yl)amino]cyclobutyl}propane-1-sulfonamide (PF-04965842): A selective JAK1 clinical candidate for the treatment of autoimmune diseases. J Med Chem.

[103] Connor CG, DeForest JC, Dietrich P, Do NM, Doyle KM, Eisenbeis S (2021). Development of a nitrene-type rearrangement for the commercial route of the JAK1 inhibitor abrocitinib. Org Process Res Dev.

[104] Lu X, Zhong Z, Zhang X (2021). Crystal forms of sulfonamide compound and preparation method.

[105] Ghosh AK, Brindisi M, Sarkar A (2018). The Curtius rearrangement: Applications in modern drug discovery and medicinal chemistry. ChemMedChem.

[106] Keam SJ (2022). Tapinarof cream 1%: First approval. Drugs.

[107] Mason AR, Mason J, Cork M, Dooley G, Hancock H (2013). Topical treatments for chronic plaque psoriasis. Cochrane Database Syst Rev.

[108] Bissonnette R, Gold LS, Rubenstein DS, Tallman AM, Armstrong A (2021). Tapinarof in the treatment of psoriasis: A review of the unique mechanism of action of a novel therapeutic aryl hydrocarbon receptor–modulating agent. J Am Acad Dermatol.

[109] Pecyna P, Wargula J, Murias M, Kucinska M (2020). More than resveratrol: New insights into stilbene-based compounds. Biomolecules.

[110] Furue M, Hashimoto-Hachiya A, Tsuji G (2019). Aryl hydrocarbon receptor in atopic dermatitis and psoriasis. Int J Mol Sci.

[111] Smith SH, Jayawickreme C, Rickard DJ, Nicodeme E, Bui T, Simmons C (2017). Tapinarof is a natural AhR agonist that resolves skin inflammation in mice and humans. J Invest Dermatol.

[112] Zhang Y (2010). Clean process for preparation of (E)-3,5-dihydroxy-4-isopropyldiphenylethene.

[113] Hoy SM (2022). Deucravacitinib: First approval. Drugs.

[114] Chimalakonda A, Burke J, Cheng L, Catlett I, Tagen M, Zhao Q (2021). Selectivity profile of the tyrosine kinase 2 inhibitor deucravacitinib compared with janus kinase 1/2/3 inhibitors. Dermatol Ther (Heidelb).

[115] Catlett I, Aras U, Liu Y, Bei D, Girgis I, Murthy B (2017). SAT0226 A first-in-human, study of BMS-986165, a selective, potent, allosteric small molecule inhibitor of tyrosine kinase 2. Ann Rheum Dis.

[116] Wrobleski ST, Moslin R, Lin S, Zhang Y, Spergel S, Kempson J (2019). Highly selective inhibition of tyrosine kinase 2 (TYK2) for the treatment of autoimmune diseases: Discovery of the allosteric inhibitor BMS-986165. J Med Chem.

[117] Roskoski R (2023). Deucravacitinib is an allosteric TYK2 protein kinase inhibitor FDA-approved for the treatment of psoriasis. Pharmacol Res.

[118] Mease PJ, Deodhar AA, van der Heijde D, Behrens F, Kivitz AJ, Neal J (2022). Efficacy and safety of selective TYK2 inhibitor, deucravacitinib, in a phase II trial in psoriatic arthritis. Ann Rheum Dis.

[119] Chen K, Deerberg J, Lin D, Dummeldinger M, Inankur B, Kolotuchin SV (2018). A process for the preparation of cyclopropanecarboxamidomethoxymethyltriazolylphenylaminomethylpyridazinecarboxamide.

[120] Matte A, Federti E, Kung C, Kosinski PA, Narayanaswamy R, Russo R (2021). The pyruvate kinase activator mitapivat reduces hemolysis and improves anemia in a β-thalassemia mouse model. J Clin Invest.

[121] Kung C, Hixon J, Kosinski PA, Cianchetta G, Histen G, Chen Y (2017). AG-348 enhances pyruvate kinase activity in red blood cells from patients with pyruvate kinase deficiency. Blood.

[122] Rab MA, Van Oirschot BA, Kosinski PA, Hixon J, Johnson K, Chubukov V (2021). AG-348 (mitapivat), an allosteric activator of red blood cell pyruvate kinase, increases enzymatic activity, protein stability, and adenosine triphosphate levels over a broad range of PKLR genotypes. Haematologica.

[123] Al-Samkari H, van Beers EJ (2021). Mitapivat, a novel pyruvate kinase activator, for the treatment of hereditary hemolytic anemias. Ther Adv Hematol.

[124] van Wijk R, van Solinge WW (2005). The energy-less red blood cell is lost: Erythrocyte enzyme abnormalities of glycolysis. Blood.

[125] Sizemore JP, Guo L, Mirmehrabi M, Su Y (2019). Preparation of amorphous and crystalline forms of N-(4-(4-(cyclopropylmethyl)piperazine-1-carbonyl)phenyl)quinoline-8-sulfonamide useful for treatment of pyruvate kinase associated disorders.

[126] Salituro FG, Saunders JO, Yan S (2010). Preparation of aroylpiperazines and related compounds as pyruvate kinase M2 modulators useful in treatment of cancer.

[127] Lamb YN (2022). Pacritinib: First approval. Drugs.

[128] Genthon A, Killian M, Mertz P, Cathebras P, Gimenez De Mestral S, Guyotat D (2021). Myelofibrosis: A review. Rev Med Interne.

[129] Hart S, Goh K, Novotny-Diermayr V, Hu C, Hentze H, Tan Y (2011). SB1518, a novel macrocyclic pyrimidine-based JAK2 inhibitor for the treatment of myeloid and lymphoid malignancies. Leukemia.

[130] Singer JW, Al-Fayoumi S, Ma H, Komrokji RS, Mesa R, Verstovsek S (2016). Comprehensive kinase profile of pacritinib, a nonmyelosuppressive janus kinase 2 inhibitor. J Exp Pharmacol.

[131] Komrokji RS, Wadleigh M, Seymour JF, Roberts AW, To LB, Zhu HJ (2011). Results of a phase 2 study of pacritinib (SB1518), a novel oral JAK2 inhibitor, in patients with primary, post-polycythemia vera, and post-essential thrombocythemia myelofibrosis. Blood.

[132] Venugopal S, Mascarenhas J (2022). The odyssey of pacritinib in myelofibrosis. Blood Adv.

[133] Singer J, Al-Fayoumi S, Ma H, Komrokji RS, Mesa R, Verstovsek S (2014). Comprehensive kinase profile of pacritinib, a non-myelosuppressive JAK2 kinase inhibitor in phase 3 development in primary and post ET/PV myelofibrosis. Blood.

[134] Mascarenhas J (2022). Pacritinib for the treatment of patients with myelofibrosis and thrombocytopenia. Expert Rev Hematol.

[135] Blanchard S, Lee CHA, Nagaraj HKM, Poulsen AS, Eric T, Tan YLE, William AD (2007). Preparation of oxygen linked pyrimidine macrocyclic derivatives as antiproliferative agents.

[136] William AD, Lee ACH, Blanchard S, Poulsen A, Teo EL, Nagaraj H (2011). Discovery of the macrocycle 11-(2-pyrrolidin-1-yl-ethoxy)-14,19-dioxa-5,7,26-triaza-tetracyclo[19.3.1.1(2,6).1(8,12)]heptacosa-1(25),2(26),3,5,8,10,12(27),16,21,23-decaene (SB1518), a potent janus kinase 2/fms-like tyrosine kinase-3 (JAK2/FLT3) inhibitor for the treatment of myelofibrosis and lymphoma. J Med Chem.

[137] Fang H, Lu H, Hou X, Yang X (2022). Preparation method of JAK inhibitor pacritinib.

[138] Fallah J, Agrawal S, Gittleman H, Fiero MH, Subramaniam S, John C (2023). FDA approval summary: Iutetium Lu 177 vipivotide tetraxetan for patients with metastatic castration-resistant prostate cancer. Clin Cancer Res.

[139] Liu X, Fang GC, Lu H, Shi ZD, Chen ZS, Han CH (2023). Lutetium Lu 177 vipivotide tetraxetan for prostate cancer. Drugs Today (Barc).

[140] Tateishi U (2020). Prostate-specific membrane antigen (PSMA)–ligand positron emission tomography and radioligand therapy (RLT) of prostate cancer. Jpn J Clin Oncol.

[141] Emmett L, Willowson K, Violet J, Shin J, Blanksby A, Lee J (2017). Lutetium 177 PSMA radionuclide therapy for men with prostate cancer: A review of the current literature and discussion of practical aspects of therapy. J Med Radiat Sci.

[142] Czernin J, Calais J (2023). The (177)Lu-PSMA-617 (Pluvicto) supply problem will be solved by competition. J Nucl Med.

[143] Chandran E, Figg WD, Madan R (2022). Lutetium-177-PSMA-617: A vision of the future. Cancer Biol Ther.

[144] Keam SJ (2022). Lutetium Lu 177 vipivotide tetraxetan: First approval. Mol Diagn Ther.

[145] Benesova M, Schafer M, Bauder-Wust U, Afshar-Oromieh A, Kratochwil C, Mier W (2015). Preclinical evaluation of a tailor-made DOTA-conjugated PSMA inhibitor with optimized linker moiety for imaging and endoradiotherapy of prostate cancer. J Nucl Med.

[146] Lin KS, Benard F, Kuo HT, Zhang Z (2019). Novel radiometal-binding compounds for diagnosis or treatment of prostate specific membrane antigen-expressing cancer.

[147] Syed YY (2022). Futibatinib: First approval. Drugs.

[148] Lamarca A, Barriuso J, McNamara MG, Valle JW (2020). Molecular targeted therapies: Ready for “prime time” in biliary tract cancer. J Hepatol.

[149] Kalyukina M, Yosaatmadja Y, Middleditch MJ, Patterson AV, Smaill JB, Squire CJ (2019). TAS-120 cancer target binding: defining reactivity and revealing the first fibroblast growth factor receptor 1 (FGFR1) irreversible structure. ChemMedChem.

[150] Goyal L, Shi L, Liu LY, Fece de la Cruz F, Lennerz JK, Raghavan S (2019). TAS-120 overcomes resistance to ATP-competitive FGFR inhibitors in patients with FGFR2 fusion–positive intrahepatic cholangiocarcinomaTAS-120 efficacy in FGFR inhibitor–resistant biliary cancer. Cancer Discov.

[151] Sootome H, Fujita H, Ito K, Ochiiwa H, Fujioka Y, Ito K (2020). Futibatinib is a novel irreversible FGFR 1–4 inhibitor that shows selective antitumor activity against FGFR-deregulated tumors irreversible FGFR1–4 inhibitor. Cancer Res.

[152] Rizzo A, Ricci AD, Brandi G (2021). Futibatinib, an investigational agent for the treatment of intrahepatic cholangiocarcinoma: Evidence to date and future perspectives. Expert Opin Investig Drugs.

[153] Futibatinib (Lytgobi) for cholangiocarcinoma. Med Lett Drugs Ther. 2023;65(1674):e69-e70. 10.58347/tml.2023.1674f. 10.58347/tml.2023.1674f 37039618

[154] Kondo M (2020). Method for producing dimethoxybenzene compound.

[155] Newell LF, Cook RJ (2021). Advances in acute myeloid leukemia. BMJ.

[156] Olutasidenib (Rezlidhia) for acute myeloid leukemia. Med Lett Drugs Ther. 2023;65(1673):e58-e59. 10.58347/tml.2023.1673e. 10.58347/tml.2023.1673e 37020343

[157] Dang L, Jin S, Su SM (2010). IDH mutations in glioma and acute myeloid leukemia. Trends Mol Med.

[158] Ward PS, Patel J, Wise DR, Abdel-Wahab O, Bennett BD, Coller HA (2010). The common feature of leukemia-associated IDH1 and IDH2 mutations is a neomorphic enzyme activity converting alpha-ketoglutarate to 2-hydroxyglutarate. Cancer Cell.

[159] de Nigris F, Ruosi C, Napoli C (2021). Clinical efficiency of epigenetic drugs therapy in bone malignancies. Bone.

[160] Jones RL, Macarulla T, Charlson JA, Van Tine BA, Goyal L, Italiano A (2020). A phase Ib/II study of olutasidenib in patients with relapsed/refractory IDH1 mutant solid tumors: Safety and efficacy as single agent. J Clin Oncol.

[161] De La Fuente MI, Colman H, Rosenthal M, Van Tine BA, Levaci D, Walbert T (2020). A phase Ib/II study of olutasidenib in patients with relapsed/refractory IDH1 mutant gliomas: Safety and efficacy as single agent and in combination with azacitidine. J Clin Oncol.

[162] Caravella JA, Lin J, Diebold RB, Campbell AM, Ericsson A, Gustafson G (2020). Structure-based design and identification of FT-2102 (Olutasidenib), a potent mutant-selective IDH1 inhibitor. J Med Chem.

[163] Dhillon S (2023). Adagrasib: First approval. Drugs.

[164] O'Sullivan É, Keogh A, Henderson B, Finn SP, Gray SG, Gately K (2023). Treatment strategies for KRAS-mutated non-small-cell lung cancer. Cancers (Basel).

[165] Adagrasib (Krazati) for NSCLC. Med Lett Drugs Ther. 2023;65(1668):e17-e18. 10.58347/tml.2023.1668f. 10.58347/tml.2023.1668f 36651795

[166] Rodenhuis S, Boerrigter L, Top B, Slebos RJ, Mooi WJ, van't Veer L (1997). Mutational activation of the K-ras oncogene and the effect of chemotherapy in advanced adenocarcinoma of the lung: A prospective study. J Clin Oncol.

[167] Hallin J, Engstrom LD, Hargis L, Calinisan A, Aranda R, Briere DM (2020). The KRASG12C inhibitor MRTX849 provides insight toward therapeutic susceptibility of KRAS-Mutant cancers in mouse models and patientstherapeutic insight from the KRASG12C inhibitor MRTX849. Cancer Discov.

[168] Ou SHI, Jänne PA, Leal TA, Rybkin II, Sabari JK, Barve MA (2022). First-in-human phase I/IB dose-finding study of adagrasib (MRTX849) in patients with advanced KRASG12C solid tumors (KRYSTAL-1). J Clin Oncol.

[169] Fell JB, Fischer JP, Baer BR, Blake JF, Bouhana K, Briere DM (2020). Identification of the clinical development candidate MRTX849, a covalent KRASG12C inhibitor for the treatment of cancer. J Med Chem.

[170] Vishwanatha T, Panguluri NR, Sureshbabu VV (2013). Propanephosphonic acid anhydride (T3P®)-A benign reagent for diverse applications inclusive of large-scale synthesis. Synthesis.

[171] Keam SJ (2022). Mavacamten: First approval. Drugs.

[172] Lakdawala N, Saberi S, Day S, Ingles J, Semsarian C, Olivotto I (2022). New York Heart Association functional class and mortality in obstructive hypertrophic cardiomyopathy. J Card Fail.

[173] Dalo JD, Weisman ND, White CM (2023). Mavacamten, a first-in-class cardiac myosin inhibitor for obstructive hypertrophic cardiomyopathy. Ann Pharmacother.

[174] Fukuda N, Granzier H, Ishiwata S, Morimoto S (2023). Editorial: Recent advances on myocardium physiology, volume II. Front Physiol.

[175] Gollapudi SK, Ma W, Chakravarthy S, Combs AC, Sa N, Langer S (2021). Two classes of myosin inhibitors, para-nitroblebbistatin and mavacamten, stabilize β-cardiac myosin in different structural and functional states. J Mol Biol.

[176] Green EM, Wakimoto H, Anderson RL, Evanchik MJ, Gorham JM, Harrison BC (2016). A small-molecule inhibitor of sarcomere contractility suppresses hypertrophic cardiomyopathy in mice. Science.

[177] Stern JA, Markova S, Ueda Y, Kim JB, Pascoe PJ, Evanchik MJ (2016). A small molecule inhibitor of sarcomere contractility acutely relieves left ventricular outflow tract obstruction in feline hypertrophic cardiomyopathy. PLoS One.

[178] Kawas RF, Anderson RL, Ingle SRB, Song Y, Sran AS, Rodriguez HM (2017). A small-molecule modulator of cardiac myosin acts on multiple stages of the myosin chemomechanical cycle. J Biol Chem.

[179] Capilupi MJ, Frishman WH (2023). Mavacamten: A novel disease-specific treatment for hypertrophic cardiomyopathy. Cardiol Rev.

[180] Oslob J, Anderson R, Aubele D, Evanchik M, Fox JC, Kane B (2014). Preparation of pyrimidinedione compounds for treating hypertrophic cardiomyopathy (HCM) and other cardiac conditions.

[181] Singal AK, Jalan R (2023). Terlipressin for hepatorenal syndrome: Opportunities and challenges. Lancet Gastroenterol Hepatol.

[182] Habas E, Ibrahim AR, Moursi MO, Shraim BA, Elgamal ME, Elzouki AN (2022). Update on hepatorenal syndrome: Definition, pathogenesis, and management. Arab J Gastroenterol.

[183] O'Brien A, Clapp L, Singer M (2002). Terlipressin for norepinephrine-resistant septic shock. Lancet.

[184] Zhou X, Tripathi D, Song T, Shao L, Han B, Zhu J (2018). Terlipressin for the treatment of acute variceal bleeding: a systematic review and meta-analysis of randomized controlled trials. Medicine.

[185] Liu X, Luo G, Jiang J, Ma T, Lin X, Jiang L (2016). Signaling through hepatocyte vasopressin receptor 1 protects mouse liver from ischemia-reperfusion injury. Oncotarget.

[186] Jao YTFN (2016). Refractory torsade de pointes induced by terlipressin (Glypressin). Int J Cardiol.

[187] Liu ZM, Zhang XY, Chen J, Shen JT, Jiang ZY, Guan XD (2017). Terlipressin protects intestinal epithelial cells against oxygen-glucose deprivation/re-oxygenation injury via the phosphatidylinositol 3-kinase pathway. Exp Ther Med.

[188] Pesaturo AB, Jennings HR, Voils SA (2006). Terlipressin: Vasopressin analog and novel drug for septic shock. Ann Pharmacother.

[189] Allegretti AS, Subramanian RM, Francoz C, Olson JC, Cárdenas A (2022). Respiratory events with terlipressin and albumin in hepatorenal syndrome: A review and clinical guidance. Liver Int.

[190] Leone M, Charvet A, Delmas A, Albanèse J, Martin C, Boyle WA (2005). Terlipressin: A new therapeutic for calcium-channel blockers overdose. J Crit Care.

[191] Chen Y, Mi P, Tao A, Yuan J (2020). Synthesis method for terlipressin.

[192] Marckmann P, Skov L, Rossen K, Dupont A, Damholt MB, Heaf JG (2006). Nephrogenic systemic fibrosis: Suspected causative role of gadodiamide used for contrast-enhanced magnetic resonance imaging. J Am Soc Nephrol.

[193] Sadowski EA, Bennett LK, Chan MR, Wentland AL, Garrett AL, Garrett RW (2007). Nephrogenic systemic fibrosis: Risk factors and incidence estimation. Radiology.

[194] Daftari Besheli L, Aran S, Shaqdan K, Kay J, Abujudeh H (2014). Current status of nephrogenic systemic fibrosis. Clin Radiol.

[195] Robic C, Port M, Rousseaux O, Louguet S, Fretellier N, Catoen S (2019). Physicochemical and pharmacokinetic profiles of gadopiclenol: A new macrocyclic gadolinium chelate with high T1 relaxivity. Invest Radiol.

[196] Bendszus M, Roberts D, Kolumban B, Meza JA, Bereczki D, San-Juan D (2020). Dose finding study of gadopiclenol, a new macrocyclic contrast agent, in MRI of central nervous system. Invest Radiol.

[197] Fries P, Massmann A, Robert P, Corot C, Laschke MW, Schneider G (2019). Evaluation of gadopiclenol and P846, 2 high-relaxivity macrocyclic magnetic resonance contrast agents without protein binding, in a rodent model of hepatic metastases: Potential solutions for improved enhancement at ultrahigh field strength. Invest Radiol.

[198] Lancelot E, Raynaud JS, Desché P (2020). Current and future MR contrast agents: Seeking a better chemical stability and relaxivity for optimal safety and efficacy. Invest Radiol.

[199] Port M (2007). Compounds comprising short aminoalcohol chains and metal complexes for medical imaging.

[200] Duggan S (2018). Omidenepag isopropyl ophthalmic solution 0.002%: First global approval. Drugs.

[201] Matsuo M, Matsuoka Y, Tanito M (2022). Efficacy and patient tolerability of omidenepag isopropyl in the treatment of glaucoma and ocular hypertension. Clin Ophthalmol.

[202] Iwamura R, Tanaka M, Okanari E, Kirihara T, Odani-Kawabata N, Shams N (2018). Identification of a selective, non-prostanoid EP2 receptor agonist for the treatment of glaucoma: omidenepag and its prodrug omidenepag isopropyl. J Med Chem.

[203] Kirihara T, Taniguchi T, Yamamura K, Iwamura R, Yoneda K, Odani-Kawabata N (2018). Pharmacologic characterization of omidenepag isopropyl, a novel selective EP2 receptor agonist, as an ocular hypotensive agent. Invest Ophthalmol Vis Sci.

[204] Nakamura N, Honjo M, Yamagishi R, Igarashi N, Sakata R, Aihara M (2021). Effects of selective EP2 receptor agonist, omidenepag, on trabecular meshwork cells, Schlemm's canal endothelial cells and ciliary muscle contraction. Sci Rep.

[205] Fuwa M, Toris CB, Fan S, Taniguchi T, Ichikawa M, Odani-Kawabata N (2018). Effects of a novel selective EP2 receptor agonist, omidenepag isopropyl, on aqueous humor dynamics in laser-induced ocular hypertensive monkeys. J Ocul Pharmacol Th.

[206] Aihara M, Lu F, Kawata H, Iwata A, Odani-Kawabata N, Shams NK (2020). Omidenepag isopropyl versus latanoprost in primary open-angle glaucoma and ocular hypertension: The phase 3 AYAME study. Am J Ophthalmol.

[207] Lamorte L, Titolo S, Lemke CT, Goudreau N, Mercier JF, Wardrop E (2013). Discovery of novel small-molecule HIV-1 replication inhibitors that stabilize capsid complexes. Antimicrob Agents Chemother.

[208] Imaizumi T, Akaiwa M, Abe T, Nigawara T, Koike T, Satake Y (2022). Discovery and biological evaluation of 1-{2,7-diazaspiro[3.5]nonan-2-yl}prop-2-en-1-one derivatives as covalent inhibitors of KRAS G12C with favorable metabolic stability and anti-tumor activity. Bioorg Med Chem.

