# Supplementary material for: Synthesis and clinical application of new drugs approved by FDA in 2022
Source: Mol Biomed. 2023 Sep 4;4:26. doi: 10.1186/s43556-023-00138-y (PMC10475455; doi:10.1186/s43556-023-00138-y)
Supplement: Supplementary file 1 — Additional file 1. [file 43556_2023_138_MOESM1_ESM.docx]

**Supporting Information**

**Synthesis and Clinical Application of New Drugs Approved by FDA in 2022**

Jing-Yi Zhang ^a^, Ya-Tao Wang ^b,c^, Lu Sun ^c,d,^*, Sai-Qi Wang^c,^*, and Zhe-Sheng Chen ^e,*^

^a^ College of Chemistry and Chemical Engineering, Zhengzhou Normal University, Zhengzhou 450044, China;

^b^ First People's Hospital of Shangqiu, Henan Province, Shangqiu 476100, China;

^c^ The Affiliated Cancer Hospital of Zhengzhou University & Henan Cancer Hospital, Henan Engineering Research Center of Precision Therapy of Gastrointestinal Cancer, Zhengzhou Key Laboratory for Precision Therapy of Gastrointestinal Cancer, Zhengzhou 450008, China;

^d^ Zhongshan Hospital Affiliated to Dalian University, Dalian 116001, China;

^e^ College of Pharmacy and Health Sciences, St. John's University, Queens, NY, 11439, USA;

*Corresponding author

Lu Sun (844039788@qq.com)

Zhe-Sheng Chen ([chenz@stjohns.edu](mailto:chenz@stjohns.edu))

Sai-Qi Wang (zlyywangsq3925@zzu.edu.cn)

| Table S1 Novel drug approvals for 2022. | | | | | |
| --- | --- | --- | --- | --- | --- |
| **No.** | **Drug Name** | **Company** | **Active Ingredient** | **Approval Date** | **FDA-Approved Use on Approval Date** |
| 1. | [Quviviq](https://www.accessdata.fda.gov/drugsatfda_docs/label/2022/214985s000lbl.pdf) | Idorsia | Daridorexant | 1/7/2022 | To treat insomnia |
| 2. | [Cibinqo](https://www.accessdata.fda.gov/scripts/cder/daf/index.cfm?event=overview.process&ApplNo=213871) | Pfizer | Abrocitinib | 1/14/2022 | To treat refractory, moderate-to-severe atopic dermatitis |
| 3. | [Kimmtrak](https://www.accessdata.fda.gov/drugsatfda_docs/label/2022/761228s000lbl.pdf) | Immunocore Ltd/Medison Pharma Ltd | Tebentafusp-tebn | 1/25/2022 | To treat unresectable or metastatic uveal melanoma |
| 4. | [Vabysmo](https://www.accessdata.fda.gov/drugsatfda_docs/label/2022/761235s000lbl.pdf) | Roche | Faricimab-svoa | 1/28/2022 | To treat neovascular (wet) aged-related macular degeneration and diabetic macular edema |
| 5. | [Enjaymo](https://www.accessdata.fda.gov/drugsatfda_docs/label/2022/761164s000lbl.pdf?utm_medium=email&utm_source=govdelivery) | Sanofi | Sutimlimab-jome | 2/4/2022 | To decrease the need for red blood cell transfusion due to hemolysis in cold agglutinin disease |
| 6. | [Pyrukynd](https://www.accessdata.fda.gov/drugsatfda_docs/label/2022/216196s000lbl.pdf) | Agios | Mitapivat | 2/17/2022 | To treat hemolytic anemia in pyruvate kinase deficiency |
| 7. | [Vonjo](https://www.accessdata.fda.gov/drugsatfda_docs/label/2022/208712s000lbl.pdf) | CTI Biopharma | Pacritinib | 2/28/2022 | To treat intermediate or high-risk primary or secondary myelofibrosis in adults with low platelets |
| 8. | [Ztalmy](https://www.accessdata.fda.gov/drugsatfda_docs/label/2022/215904s000lbl.pdf) | Marinus | Ganaxolone | 3/18/2022 | To treat seizures in cyclin-dependent kinase-like 5 deficiency disorder |
| 9. | [Opdualag](https://www.accessdata.fda.gov/drugsatfda_docs/label/2022/761234s000lbl.pdf) | Bristol-Myers Squibb | Nivolumab and relatlimab-rmbw | 3/18/2022 | To treat unresectable or metastatic melanoma |
| 10. | [Pluvicto](https://www.accessdata.fda.gov/scripts/cder/daf/index.cfm?event=overview.process&ApplNo=215833) | Novartis | Lutetium (^177^Lu) vipivotide tetraxetan | 3/23/2022 | To treat prostate-specific membrane antigen-positive metastatic castration-resistant prostate cancer following other therapies |
| 11. | [Vivjoa](https://www.accessdata.fda.gov/drugsatfda_docs/label/2022/215888s000lbl.pdf) | Mycovia | Oteseconazole | 4/26/2022 | To reduce the incidence of recurrent vulvovaginal candidiasis (RVVC) in females with a history of RVVC who are not of reproductive potential |
| 12. | [Camzyos](https://www.accessdata.fda.gov/drugsatfda_docs/label/2022/214998s000lbl.pdf) | Bristol-Myers Squibb | Mavacamten | 4/28/2022 | To treat certain classes of obstructive hypertrophic cardiomyopathy |
| 13. | [Voquezna](https://www.accessdata.fda.gov/scripts/cder/daf/index.cfm?event=overview.process&ApplNo=215152) | Phathom | Vonoprazan, amoxicillin, and clarithromycin | 5/3/2022 | To treat *Helicobacter pylori* infection |
| 14. | [Mounjaro](https://www.accessdata.fda.gov/scripts/cder/daf/index.cfm?event=overview.process&ApplNo=215866) | Eli Lilly | Tirzepatide | 5/13/2022 | To improve blood sugar control in diabetes, in addition to diet and exercise |
| 15. | [Vtama](https://www.accessdata.fda.gov/drugsatfda_docs/label/2022/215272s000lbl.pdf) | Dermavant | Tapinarof | 5/23/2022 | To treat plaque psoriasis |
| 16. | [Amvuttra](https://www.accessdata.fda.gov/drugsatfda_docs/label/2022/215515s000lbl.pdf) | Alnylam | Vutrisiran | 6/13/2022 | To treat polyneuropathy of hereditary transthyretin-mediated amyloidosis |
| 17. | [Xenpozyme](https://www.accessdata.fda.gov/drugsatfda_docs/label/2022/761261s000lbl.pdf) | Sanofi | Olipudase alfa | 8/31/2022 | To treat Acid Sphingomyelinase Deficiency [Press Release](https://www.fda.gov/news-events/press-announcements/fda-approves-first-treatment-acid-sphingomyelinase-deficiency-rare-genetic-disease) |
| 18. | [Spevigo](https://www.accessdata.fda.gov/drugsatfda_docs/label/2022/761244s000lbl.pdf) | Boehringer-Ingelheim | Spesolimab-sbzo | 9/1/2022 | To treat generalized pustular psoriasis flares |
| 19. | [Daxxify](https://www.accessdata.fda.gov/drugsatfda_docs/label/2022/761127s000lbl.pdf) | Revance Therapeutics | DaxibotulinumtoixnA-lanm | 9/7/2022 | To treat moderate-to-severe glabellar lines associated with corrugator and/or procerus muscle activity |
| 20. | [Sotyktu](https://www.accessdata.fda.gov/drugsatfda_docs/label/2022/214958s000lbl.pdf) | Bristol-Myers Squibb | Deucravacitinib | 9/9/2022 | To treat moderate-to-severe plaque psoriasis |
| 21. | [Rolvedon](https://www.accessdata.fda.gov/drugsatfda_docs/label/2022/761148Orig1s000Corrected_lbl.pdf) | Spectrum Pharmaceuticals | Eflapegrastim | 9/9/2022 | To decrease the incidence of infection in patients with non-myeloid malignancies receiving myelosuppressive anti-cancer drugs associated with clinically significant incidence of febrile neutropenia |
| 22. | [Terlivaz](https://www.accessdata.fda.gov/drugsatfda_docs/label/2022/022231s000lbl.pdf) | Mallinckrodt | Terlipressin | 9/14/2022 | To improve kidney function in adults with hepatorenal syndrome with rapid reduction in kidney function |
| 23. | [Elucirem](https://www.accessdata.fda.gov/drugsatfda_docs/label/2022/216986s000lbl.pdf) | Guerbet | Gadopiclenol | 9/21/2022 | To detect and visualize lesions, together with MRI, with abnormal vascularity in the central nervous system and the body |
| 24. | [Omlonti](https://www.accessdata.fda.gov/drugsatfda_docs/label/2022/215092s000lbl.pdf) | Santen Pharmaceutical/UBE Corporation | Oomidenepag isopropyl ophthalmic solution | 9/22/2022 | To reduce elevated intraocular pressure in patients with open‑angle glaucoma or ocular hypertension |
| 25. | [Relyvrio](https://www.accessdata.fda.gov/drugsatfda_docs/label/2022/216660s000lbledt.pdf) | Amylyx Pharmaceuticals | Sodium phenylbutyrate/taurursodiol | 9/29/2022 | To treat amyotrophic lateral sclerosis (ALS) [Press Release](https://www.fda.gov/news-events/press-announcements/fda-approves-new-treatment-option-patients-als) |
| 26. | [Lytgobi](https://www.accessdata.fda.gov/drugsatfda_docs/label/2022/214801Orig1s000lbledt.pdf) | Taiho Pharmaceutical | Futibatinib | 9/30/2022 | To treat intrahepatic cholangiocarcinoma harboring fibroblast growth factor receptor 2 (FGFR2) gene fusions or other rearrangements |
| 27. | Imjudo | AstraZeneca | Tremelimumab | 10/21/2022 | To treat unresectable hepatocellular carcinoma |
| 28. | Tecvayli | Johnson & Johnson | Teclistamab-cqyv | 10/25/2022 | To treat relapsed or refractory multiple myeloma among adults who have received at least four specific lines of therapy |
| 29. | Elahere | ImmunoGen | Mirvetuximab Soravtansine-gynx | 11/14/2022 | To treat patients with recurrent ovarian cancer that is resistant to platinum therapy |
| 30. | Tzield | Provention Bio (PRVB.US) | Teplizumab-mzwv | 11/18/2022 | To delay the onset of stage 3 type 1 diabetes |
| 31 | Rezlidhia | Rigel Pharmaceuticals | Olutasidenib | 12/01/2022 | To treat adults with relapsed or refractory acute myeloid leukemia with a susceptible isocitrate dehydrogenase-1 (IDH1) mutation |
| 32 | Krazati | Mirati Therapeutics | Adagrasib | 12/12/2022 | To treat KRAS G12C-mutated locally advanced or metastatic non-small cell lung cancer in adults who have received at least one prior systemic therapy |
| 33 | Sunlenca | Gilead Sciences | Lenacapavir | 12/22/2022 | To treat adults with HIV whose HIV infections cannot be successfully treated with other available treatments due to resistance, intolerance, or safety considerations |
| 34 | Lunsumio | Genentech | Mosunetuzumab-axgb | 12/22/2022 | To treat adults with relapsed or refractory follicular lymphoma, a type of non-Hodgkin lymphoma |
| 35 | Xenoview | Polarean | Hyperpolarized Xe-129 | 12/23/2022 | To evaluate pulmonary function and imaging |
| 36 | Briumvi | TG Therapeutics | Ublituximab-xiiy | 12/28/2022 | To treat relapsing forms of multiple sclerosis |
| 37 | NexoBrid | MediWound | Anacaulase-bcdb | 12/28/2022 | To remove eschar in adults with deep partial thickness or full thickness thermal burns |
